# A transdiagnostic conflict-square algorithm: a four-node computational framework for psychotherapy and functional diagnosis

**DOI:** 10.3389/fpsyt.2026.1687372

**Published:** 2026-03-16

**Authors:** Eik Niederlohmann

**Affiliations:** Department of Psychosomatic Medicine and Psychotherapy, Kliniken Erlabrunn, Breitenbrunn, Germany

**Keywords:** computational psychiatry, computational psychotherapy, psychotherapy process coding, conflict-square algorithm, human-in-the-loop, ICD-11 severity, mini-ICF-APP, Perceive-Assess-Dose-Safeguard (PAD-S)

## Abstract

Clinical services need more than categorical labels to guide safe dosing, maintain alliance, and plan functional recovery. The Conflict-Square Algorithm (CSA) offers a compact bedside grammar for moment-to-moment decisions during psychotherapy. Clinicians track four observable signals—defense, anxiety/affect tolerance, progression, and superego/shame—and gate intervention intensity by three safety thresholds (A–C). Each clinically meaningful moment is summarized as one auditable episode line in plain language: trigger, observable response, threshold, action, and expected functional impact (Mini-ICF-APP). To strengthen reproducibility, we provide i) operational threshold definitions with observable markers and common misclassification errors, grounded in established anxiety-channel descriptions [striated muscle, smooth muscle, and cognitive-perceptual disruption (CPD)]; ii) a short scope and contraindication checklist; iii) several consecutive worked micro-episodes demonstrating node shifts, threshold transitions, and dose modulation over time; and iv) a minimal machine-readable schema plus a threshold-gating state diagram. As a proof-of-concept feasibility demonstration, we report aggregate coding statistics from three published psychotherapy training videos distributed by the ISTDP Institute (transcribed for analysis with written permission; N = 2,809 speaker turns) using a three-label therapist intervention mapping (invite progression/defense work/anxiety regulation) aligned with CSA nodes. CSA is presented as a teachable, testable decision framework—not as a validated diagnostic instrument—and we outline a pragmatic validation program (rater agreement, safety-rule adherence, usability, and functional outcomes) and future multimodal extensions (e.g., optional physiological monitoring for biofeedback and threshold detection).

## 1 Introduction

Psychiatric categories remain indispensable for communication, reimbursement, and research coordination, yet at the point of care, they leave crucial questions unanswered: who destabilizes under interpersonal load, which level of care is both safe and efficient, and how quickly (and under what conditions) can everyday roles be resumed? The recent shift toward dimensional severity and impairment in the Diagnostic and Statistical Manual of Mental Disorders, Fifth Edition (DSM‑5) Alternative Model for Personality Disorders (AMPD) and in International Classification of Diseases, 11th Revision (ICD‑11) acknowledges these gaps, but categorical codes still compress heterogeneous structural capacities and therefore weakly predict in-session tolerance, destabilization risk, or the safe dose of intervention (1–4). Transdiagnostic evidence further suggests that shared mechanisms cut across categories, reinforcing the need to represent capacities under stress rather than only labels (5). This paper proposes a compact, teachable, and computationally tractable adjunct—the Conflict-Square Algorithm (CSA)—that augments categorical diagnosis with *in vivo* performance and links observations to the shared language of functioning used across services (6–11). The CSA formalizes psychotherapy as a controlled relational stress test organized around four observable decision nodes—defense, anxiety/affect tolerance, progression (any forward relational movement), and superego/shame—and three safety thresholds that gate intervention intensity. Each clinically meaningful interaction is documented as a brief episode with five fields in words: trigger, observable response, threshold (A, B, or C), action, and functional impact. This grammar yields standardized information for clinical handoffs, training, and human-in-the-loop computational support while prioritizing safety heuristics such as stopping deepening at signs of cognitive-perceptual disruption and protecting positive affect when it appears (12–20). All procedural rules are presented in continuous prose; material previously in tables has been integrated into the narrative; a single cohesive clinical case illustrates pacing, alliance repair, and functional goal-setting without turning the encounter into a checklist.

*Terminology note*. The label “Conflict Square” was chosen to keep an explicit clinical lineage to the triangle-of-conflict tradition in experiential-dynamic therapies while extending it with i) a dedicated superego/shame node for post-progress punitive self-attack and ii) A–C safety thresholds that gate intervention intensity. In subsequent work, the same core four-node/three-threshold logic has been reframed as the PAD-S (Perceive–Assess–Dose–Safeguard) decision matrix to provide a more school-neutral naming and an explicit state–action representation optimized for annotation, supervision workflows, and computational modeling. CSA and PAD-S should therefore be read as complementary notational layers for the same underlying process grammar (clinician-facing prose documentation *vs*. machine-readable decision matrix); for the PAD-S formulation and an serious mental illness (SMI)-focused implementation pathway, see Niederlohmann (2026) (21).

### 1.1 From categories to capacities

ICD-11’s severity-first stance—most visible in the personality disorder chapter—creates an entry point for a capacity-oriented formulation: what changes when minimal, meaningful relational load is introduced, and at which threshold do symbolization and participation falter (3, 4, 22, 23)? In practice, clinicians titrate the dose of intervention from observable tolerance and failure modes—shifts between anxiety pathways, defensive detours, and shame-driven collapses—rather than from labels alone. The CSA makes those micro-observations explicit and portable by defining three thresholds that function as safety gates. Threshold A denotes regulated arousal with intact symbolization and room to deepen; threshold B denotes a workable but narrowing window that calls for brief exposures, frequent regulation, and explicit check-ins; threshold C denotes cognitive-perceptual disruption or a shame collapse that mandates de-escalation and re-entry only once A–B stability is restored. Because thresholds are judged from concrete markers—skeletal tension and breath-holding for striated-muscle anxiety, upper-abdominal cramp or nausea for smooth-muscle activation, and fogging or derealization for cognitive-perceptual disruption—dose control becomes observable, teachable, and auditable rather than tacit (16–18, 24). Function is the organizing outcome. Each episode closes with a functional impact line expressed in Mini-ICF-APP terms—endurance/persistence, decision making, planning/structuring, rule adherence, assertiveness, dyadic relatedness, and group interaction—with a concrete target and a re-check interval (6–11). This structure-to-severity-to-function chain clarifies why individuals who share the same category may justifiably receive different safeguards, timelines, or resources: their capacities and failure points under load are not equivalent. The emphasis on dosing to the window of tolerance and on protecting nascent adaptive states is consistent with psychodynamic–experiential pedagogy, including intensive short-term dynamic psychotherapy (ISTDP) and affect-phobia therapy, where observable micro-markers determine whether to deepen affect, regulate, or buffer positives before further work (12–18, 25).

### 1.2 The CSA in one sentence: rendered in prose

At each decision point, the clinician identifies the leading process in the room (defense, anxiety/affect tolerance, progression, or superego/shame), judges the current threshold (A, B, or C), selects standard versus graded dosing accordingly, protects positive affect when it appears, and documents trigger, response, threshold, action, and functional impact in a single sentence. These fields map cleanly to data variables (node, threshold, anxiety pathway, defense band, presence of superego attack, action taken, and Mini-ICF-APP target), supporting inter-rater training and digital quality assurance. Where helpful, the clinical loop can be read through the lens of active inference: therapy becomes an iterated perception–action cycle in which small, safe prediction errors drive learning, explicit thresholds that stabilize updates, and surface indicators of threshold transitions for supervisory attention (language and paralinguistics) without dictating care (19, 20, 26–31). See **Appendix A** for the interpretive map.

### 1.3 Contribution and relevance

Clinically, the CSA clarifies when to block specific tactical defenses, when to regulate, when to deliver brief exposures near a narrowing window, when to protect positive affect from a punitive process, and when to halt deepening altogether. This reduces inadvertent crossings into destabilization, supports alliance by matching dose to tolerance and buffering joy states, and ties each micro-decision to functional targets that teams can monitor across settings (8, 10, 12–18). Computationally, standardized episode lines yield interpretable labels for human-in-the-loop pipelines: lexical and paralinguistic features associated with threshold transitions or post-progress superego attacks can be surfaced for supervisory attention, fidelity feedback, and reflective practice without imposing real-time coding rituals or black-box automation (29, 30, 32). Administratively and forensically, because outputs are already phrased in Mini-ICF-APP language and linked to severity-anchored thresholds, they support stepped-care triage, level-of-care justification, and transparent documentation of disability and progress; service models such as track-based pathways and recovery-oriented reforms provide additional implementation anchors (7, 8, 10, 33, 34). For training and supervision, the compact vocabulary—four nodes, three thresholds, and a small set of actions—lends itself to short drills, video-based deliberate practice, and structured feedback, with emerging trials underscoring the value of deliberate practice formats for interpersonal skill acquisition (35, 36). The broader evidence base for short-term dynamic and experiential work, including outcome syntheses and cost-effectiveness data, provides plausibility for a safety-and-dose framework that abstracts shared rules into school-neutral prose and functional targets, while acknowledging its roots in experiential-dynamic/ISTDP training traditions (12–18, 37, 38).

### 1.4 Scope and positioning of the CSA

The CSA is advanced as a conceptual and algorithmic framework with a worked clinical illustration; it is not presented as a validated diagnostic instrument or as a directive for real-time coding during sessions. Its primary domain is training, supervision, and quality improvement, where it standardizes observation, structures deliberate-practice routines, and offers a transparent decision grammar that can, with clinician consent and control, inform assistive analytics. In live sessions, application is appropriate when thresholds and alliance can be actively maintained—work proceeds within A–B, graded exposures are used near a drift from B to C, and positive states are protected before any further deepening. In contexts marked by severe fragility, rapid transitions to cognitive-perceptual disruption, or acute inpatient pressures, a training-first stance is prudent, with any in-session use limited to safety-oriented prompts and alliance repair (8, 16–18). The clinical case later in the paper demonstrates how an episode grammar guides pacing and functional goal-setting without collapsing the encounter into a checklist, while a staged validation agenda—inter-rater reliability for nodes and thresholds, adherence to dose logic, safety, and alliance outcomes, and functioning endpoints—keeps claims appropriately conservative (6–8, 10, 12–14, 35, 36). Positioned within computational psychiatry, the CSA’s crisp episodes and thresholds align with active-inference perspectives on interaction as iterative perception–action under uncertainty and provide clean, interpretable labels for clinician-supervised natural language processing (NLP) and paralinguistic flagging of safety-relevant transitions (19, 20, 26–30). In services research, phrasing decisions in Mini-ICF-APP terms facilitates stepped-care decisions and interprofessional coordination without additional paperwork burden (6–11). We next sharpen three bedside clarifications that make the rules safe and teachable (Section 2), specify the bedside routine in prose (Section 3), map it to ICD-11 and AMPD and Mini-ICF-APP (Section 4), and thread a compact clinical case through the rules (Section 5) before outlining scope and validation (Sections 6–8). Roadmap. Section 2 refines the four nodes and thresholds with safety consequences. Section 3 expresses the algorithm as a readable routine. Section 4 maps the episode grammar to ICD-11/AMPD and Mini-ICF-APP. Section 5 integrates a composite case. Sections 6 and 7 cover scope and validation. Section 8 synthesizes limits and implications.

## 2 Conceptual foundations and refinements

This section offers three clarifications that are immediately teachable at the bedside and that have direct consequences for safety, dosing, and outcome. First, we refine the superego/shame node beyond the shorthand of “shame alone”, distinguishing shame, guilt, and self-accusation and situating “subjectivizing attempts” (including delusion-like elaborations) as adaptive efforts to recover coherence after collapse. Second, we draw a clean line between defense styles (structural organization of affect regulation) and resistance (a process that emerges within the therapeutic situation). Third, we argue that anxiety and affect tolerance do not lie on a single inverse continuum; we note configurations with low observable anxiety yet pronounced affect intolerance and specify the consequences for dosing and safety. These clarifications are encoded in the CSA as four observable nodes—defense, anxiety/affect tolerance, progression, and superego/shame—gated by A–C thresholds and a small set of rule-bound micro-actions (16–18, 25, 35, 36).

### 2.1 Superego is not “just shame”: distinguishing shame, guilt, and self-accusation—and recognizing subjectivizing attempts

Why the distinction matters. Conflating shame with the broader superego system obscures clinically important differences. Shame is a global self-devaluation—”I am bad”—with characteristic posture and vocal changes and a high likelihood of “rapid reversal after a positive moment” following moments of pride or closeness. Guilt is an appraisal of transgression—”I did something bad”—and often mobilizes reparative action. Self-accusation is the content and tone of the punitive introject that attacks needs, closeness, and progress. In practice, these phenomena co-occur but call for different micro-decisions: shame states are buffered and downregulated; reparative guilt can be harnessed to agency; self-accusation is externalized and limited before any deepening. This sequencing is explicit in contemporary experiential-dynamic teaching, where clinicians protect nascent positive states and only then resume exposure or defense work (15–18). Observable markers and teachable responses. The superego/shame node is detected through lexical cues (global verdicts and moralized self-talk) and non-verbal markers (head drop, averted gaze, and postural collapse), frequently following a small progression step. A reliable response sequence is i) name the switch, ii) protect the positive state to consolidate it, iii) externalize and limit the punitive voice, and iv) re-enter graded exposure only within demonstrated tolerance. This preserves alliance, prevents C-level collapse, and consolidates gains; it is easily trained with brief prompts and supervision checklists (16–18). Persecutory superego and destructive guilt. In severe or mixed structures, a persecutory superego may masquerade as “resistance” while actually reflecting structural pathology that collapses function under minimal relational load. When global self-condemnation follows a small step forward, the correct choice is not “more pressure” but protection of the positive state, explicit limits toward the punitive process, and restoration of tolerance prior to any renewed deepening. This move reduces mis-dosing and costs little time (16–18). Subjectivizing attempts as coherence repair. In destabilized states—especially as shame escalates toward cognitive-perceptual disruption—patients may produce delusion-like elaborations that function to restore symbolic coherence after breakdown. Within active-inference terms, such narratives can be viewed as self-evidencing moves by a stressed generative model attempting to reduce intolerable prediction error by imposing structure on noisy input; regulation, protection of positives, and only then gentle reality-testing are therefore more humane and effective than debating “truth value” while tolerance is compromised (19, 20, 26, 27, 39). Summary. Superego dynamics extend beyond shame to include reparative guilt and destructive self-accusation. *In vivo*, the CSA ensures that we detect the switch that often follows progress, protect positives, externalize and limit the critic, and resume graded deepening only within tolerance (15–18). Pride share → head drop + global self-attack → B → C risk → protect positives; externalize; 2-second exposure → dyadic relatedness: one protected appreciation exchange/week; re-check 4–6 weeks.

### 2.2 Resistance versus defense: structural organization versus process in the room

Why the distinction matters. Defenses are elements of structural organization—habitual, often historically adaptive ways of modulating proximity to warded-off affects (for example, isolation of affect, rationalization, and splitting). Resistance, by contrast, is a process that emerges within the therapeutic situation: a here-and-now barrier to emotional closeness or task engagement, often crystallizing in the relationship with the therapist. Conflating the two blunts clinical precision and invites mis-dosing—either confronting structural defenses that the system cannot yet tolerate or reifying live resistance as a fixed trait (13, 14, 16–18, 25). Operational consequences in CSA. When defense is in front of the system and tolerance is stable (A–B; striated-muscle anxiety), clinicians block specific tactical defenses; link defense to feeling, wish, and action; and deepen. When tolerance narrows toward B–C (smooth-muscle activation or early cognitive-perceptual signs), partial blocking with regulation and seconds-long exposures replaces pressure. When resistance to closeness is in front of the system (icy compliance, covert opposition, and relational detachment), the process in the room is addressed first—repairing strains and protecting positive states—before any confrontation of defenses. This two-track logic aligns with ISTDP psychodiagnostic scaffolds and with affect-phobia pedagogy that teaches graduated exposure anchored in a window-of-tolerance (13, 14, 16–18, 25). Clinical payoff and teachability. On the ground, the distinction prevents two common errors: escalating pressure against character defenses when the real problem is a post-progress superego attack, and “working content” while the relationship has shifted into process resistance. Because node identification and thresholding are observable, these decisions are trainable and auditable in supervision and deliberate-practice formats (16–18, 35, 36).

### 2.3 Anxiety and affect tolerance are not a single inverse continuum

The tempting but unsafe assumption. It is tempting to infer that low observable anxiety implies high affect tolerance. Clinical experience and structural diagnostics contradict this. Some configurations—psychopathy-like presentations, certain perverse organizations, and splitting under attachment load—show little overt anxiety while remaining intolerant of affects that threaten structure, especially closeness, pride, or grief. Apparent calm may mask dissociation, detachment, or rapid superego attacks that derail functioning as soon as authentic affect approaches (5, 13, 16–18). What to track instead. Rather than trusting surface calm, the CSA tracks pathways (striated to smooth muscle to cognitive-perceptual), refractory periods, and drifts toward collapse as dose rises. Standard dosing is justified only when stability within A–B is demonstrated under attachment-laden probes; absent that, fragility is assumed, and proximity is micro-dosed. This rule is simple, falsifiable in supervision, and consistent with psychodiagnostic teaching (13, 16–18, 25). Active-inference rationale. Under a predictive-processing lens, low anxiety can reflect over-confident priors that suppress error signals until relational input forces a regime change, at which point collapse occurs. Carefully titrated, seconds-long exposures and precision control of attention, breath, and gaze minimize catastrophic updates and stabilize learning (19, 20, 26–28). Concrete exceptions and implications for dose. • Psychopathy-like configurations may present with contemptuous detachment and rapid punitive attacks after small gains; dose is graded, positives are protected, and limits are explicit, with functional expectations focused on rule adherence and dyadic relatedness under humiliation cues. • Perverse defensive patterns may sexualize or debase positive affect; the clinician delivers seconds-long exposure to pride or joy, consolidates the state, and introduces reality-testing only after buffering. • Splitting in mixed structures often shows a calm narrative with sudden cognitive-perceptual disruption at ambiguity; graded work from the outset, brief cycles, and narrow blocking within the B range are safer. These patterns are readily coached in brief drills and mapped to Mini-ICF-APP phrasing for team use (6–8, 16–18, 25).

### 2.4 Take-home definitions (inline call-out in prose)

*Defense (DEF)*. It is a historically adaptive coping mode (for example, isolation, rationalization, and splitting) that modulates proximity to warded-off affects. When defense is in front of the system and tolerance is stable (A–B), name and block specific tactical maneuvers and deepen; when the system shows drift toward collapse or cognitive-perceptual markers under attachment-laden stimuli, partial blocking, regulation, and micro-dosing replace pressure (13, 16–18, 25). Anxiety/affect tolerance (ANX). The physiology of load: striated-muscle tension (A–B), smooth-muscle activation (B with risk), and cognitive-perceptual disruption (C). Work is kept within B, downshifted at B–C, and stopped at C until symbolization returns. Low observable anxiety is not treated as high tolerance unless demonstrated under relationally meaningful probes (13, 16–18). Progression (PRO). It is any forward relational movement—naming a wish or need, tolerating a brief moment of contact, accepting help, or making a small value-consistent action. Validate, link to a tiny step, and avoid enlarging too quickly; protect the step from punitive dynamics before any further deepening (15–18). Superego/shame (SUP). A punitive system that attacks needs, closeness, and joy through global verdicts, contempt, and postural collapse, often immediately after progress. Name the switch, protect positives, externalize and limit the critic, and then resume graded deepening. Delusion-like elaborations in collapse states are read as coherence repair and handled by regulation and buffering prior to reality-testing (16–20, 26). A–C thresholds (window of tolerance). A: regulated arousal with intact symbolization—proceed and deepen. B: narrowing but workable—micro-dose and monitor. C: cognitive-perceptual disruption or shame collapse—stop deepening, regulate, protect positives, repair alliance, and then re-enter within A–B (13, 16–18, 25). Operational definitions and observable indicators for thresholds A–C, including common misclassification errors and safeguards, are summarized in **Table 1**. Clinical payoffs and the bridge to functioning. By tying node/threshold patterns to Mini-ICF-APP domains, the same in-session phenomena that guide dosing also predict which functions fail under load; for example, endurance and group interaction decline at C-level disruption; planning and structuring degrade when detours proliferate under closeness; rule adherence fluctuates under humiliation cues that elicit punitive dynamics. These readouts convert smoothly into copy-ready phrasing and re-check intervals for interprofessional teams and stepped-care decisions (6–8, 10, 11). Teaching and quality-assurance implications. Deliberate-practice drills built around the CSA episode sentence make these distinctions learnable and auditable; emerging training studies suggest that structured feedback improves interpersonal micro-skills, and standardized labels enable rater agreement and fidelity checks (29, 30, 35, 36). Alignment with wider literatures and computational bridges. The clarifications above are not school-bound. Affect-phobia therapy’s conflict and person triangles, ISTDP psychodiagnostics, and contemporary supervision texts converge on tracking the leading process, differentiating structural defenses from in-room resistance, protecting positive states before confrontation, and dosing to the window of tolerance with explicit stop rules. Rendering these convergences as machine-readable labels creates a natural interface for human-in-the-loop computational assistance-language and paralinguistic pipelines that flag threshold transitions or post-progress superego activation for supervision—while preserving clinician control (16–20, 25, 29, 30). One-paragraph takeaway. Superego dynamics must be parsed into shame, guilt, and self-accusation—with subjectivizing attempts read as coherence repair—to avoid over-pressing and collapsing the system; defenses (structure) are not the same as resistance (process) and call for different actions; and “low anxiety” cannot be assumed to mean high tolerance without graded proof under attachment-laden probes. Encoding these distinctions within the CSA’s four nodes and A–C thresholds yields simple, teachable, auditable rules that improve safety, alliance, and functional outcomes and that align psychodynamic craft with computational psychiatry and service realities (16–20, 25, 35, 36). These distinctions (SUP beyond shame; defense versus resistance; low anxiety ≠ high tolerance) set the guardrails for the bedside routine specified next. Bridge. The clarifications above become concrete choices in the next section’s step-by-step routine.

**Table 1:** Operational definitions for thresholds A–C (with observable indicators and common misclassification errors).

Threshold	Decision rule/definition	Observable indicators (examples)	Common misclassification errors (and safeguards)
A	Safe range or under-activated/over-regulated. Symbolization and contact are intact; work can proceed. If under-activated, the main need is mobilization (focus, specificity, and contact) rather than regulation.	Coherent speech; stable attention; mild or absent somatic anxiety. If anxiety is present, it is typically striated-muscle signaling such as hand clenching, arm/neck tension, or sighing respirations (40, 41).	Mistaking distance/compliance for safety: A can be “quiet avoidance”. Safeguard: test with small probes and check for genuine engagement, not only calmness.
B	Working range with a narrowing window. Symbolization remains intact, but load rises; dosing must be actively titrated (shorter exposures, frequent check-ins, and early regulation).	Constriction without confusion: language remains coherent but becomes terse/looping. Anxiety may shift toward smooth-muscle signaling (relative absence of striated tension with stomach/bladder symptoms) (40).	Over-calling A when smooth-muscle signs appear. Safeguard: treat smooth-muscle activation as a B-level risk signal and shift to graded format early.
C	Stop rule. Cognitive-perceptual disruption (CPD) or shame-collapse signs indicate that deepening is unsafe. De-escalate, regulate, and re-enter only after A/B stability is restored.	Loss of cognitive clarity (losing track of thoughts, blurry vision, loss of hearing or limb sensation) or conversion-like signs (weakness/paralysis), often with relative absence of striated tension (40, 41). Shame-collapse pattern: abrupt head drop, averted gaze, voice fading, and global self-condemnation immediately after progress.	Misreading CPD as “resistance” and pushing harder. Safeguard: treat CPD/shame collapse as safety-critical and switch to regulation/protection, not confrontation.

Observable anxiety-channel manifestations are summarized from **Table 1** in Abbass and Haghiri (2025) and clinical descriptions in Abbass (2015) (40, 41).

## 3 The algorithmic core in prose

This section specifies the CSA as a readable bedside routine. It describes how a therapist a) identifies the leading clinical signal, b) checks safety thresholds, c) matches the dose of intervention (standard versus graded), d) protects positive affect when punitive dynamics activate, and e) documents each micro-cycle in a single sentence that also translates into Mini-ICF-APP targets. No diagrams or symbols are required; operational detail appears throughout in natural language and is grounded in contemporary experiential-dynamic pedagogy (ISTDP and affect-phobia therapy), dimensional nosology (ICD-11 and AMPD), and an active-inference view of psychotherapy as iterated perception–action under uncertainty (13–20, 25).

### 3.1 Recognizing the front of system signal

At each conversational turn, the clinician first determines which of four observable nodes is currently in charge: defense, anxiety/affect tolerance, progression, or superego/shame. This is an orienting judgment based on ordinary language and visible markers. Defense is evident when talk detours from a concrete feeling or wish into analysis, generalization, joking, or idealization–devaluation. In robust conditions, these maneuvers can be named and briefly blocked; in fragile conditions, they are only partially blocked, while tolerance is stabilized (13, 14, 16–18, 25). Anxiety/affect tolerance is read from the pathway and trend more than intensity alone. Skeletal tension and breath-holding signal the workable range; upper gastrointestinal sensations or urgency indicate narrowing; fogging, derealization, or confused speech signal a stop point. Speech remains coherent within the working window, constricts as the window narrows, and fragments when it closes (13, 14, 16–18). Progression means any forward relational movement, such as naming a wish, tolerating a few seconds of eye contact, accepting help, or making a small, value-consistent commitment. These steps are validated and linked to tiny actions rather than being enlarged too quickly (15–18, 25). Superego/shame becomes the front of the system when global self-condemnations, contempt toward needs, head drop, averted gaze, or postural collapse appear—often immediately after a positive moment. Here, the priority is to protect the nascent positive state and externalize the punitive voice before any further deepening (16–18). The front of the system readout is spoken in plain language. When tolerance and alliance are robust, gentle blocking and deepening follow; under fragility or mixed presentations, partial blocking, co-regulation, and seconds-long exposures are safer. Two clarifications improve precision: defenses (trait-like coping styles) are distinguished from resistance (a process in the room), and low observable anxiety is not taken to mean high affect tolerance without graded proof under attachment-laden triggers (13–18, 25). An example of a cognitive-perceptual stop-rule is shown in Section 5.2, Episode 5.

### 3.2 Checking thresholds with markers of the anxiety pathways

The second step estimates the window of tolerance using three thresholds and their pathway markers. Threshold A (under-activated/regulated) means affect, and symbolization are available; skeletal tension may be present but does not impair coherence; brief eye contact is manageable. Standard deepening is typically safe. Threshold B (narrowing/working range) shows rising skeletal tension, intermittent smooth-muscle activation, shallower breathing, narrowed attention, and shorter utterances. This is the dosing zone: shorter exposures, frequent regulation, and explicit check-ins. A drift from the working range toward collapse calls for immediate dose reduction. Threshold C (cognitive-perceptual disruption or shame collapse) presents as fogging, tunnel vision, derealization, confused speech, or a sequence in which a positive state flips into collapse. Deepening stops here; contact and regulation are restored before re-entry at A or B. Pathways are described as striated muscle (typical for the working range), smooth muscle (narrowing with risk of collapse), and cognitive-perceptual (collapse). Tracking which pathway discharges and how fast it escalates makes dosing teachable and auditable and aligns with established experiential-dynamic psychodiagnostics (13, 14, 16–18, 25). An active-inference lens clarifies why these thresholds matter: safe, small prediction errors enable learning; crossing into collapse produces catastrophic updates, and alliance ruptures (19, 20, 26–28). Graded dosing near a B → C drift is illustrated in Section 5.2, Episode 3.

### 3.3 Choosing the dose (standard versus graded, micro-exposure near the boundary, and stop rules)

With node and threshold in view, the therapist matches the dose. Standard format (tolerance adequate; alliance can carry load): name and block specific tactical defenses; return to the immediate feeling toward a here-and-now target; invite brief, focused experiencing; and link any progression to a small action. If defensive crystallization becomes syntonic and characterological, increase clarity and proportionate challenge. If punitive dynamics intrude after progress, protect the positive state first and only then consider further confrontation (13, 14, 16–18). Graded format (fragility or mixed presentations; proximity to collapse): use partial blocks; introduce seconds-long exposure windows with safety scaffolds such as paced exhalation, orienting, and brief eye contact; pause early and check bodily markers; reinforce micro-progressions (for example, a 2-second steady gaze) and then stop before the boundary to collapse. Resume only after observable return to the working window (13, 14, 16–18, 25). Stop rules are simple and non-negotiable. At signs of cognitive-perceptual disruption or shame collapse, deepening stops, and regulation is restored. When a positive state is immediately attacked, the positive state is protected before any further deepening. These rules reduce inadvertent over-exposure and support alliance repair and are consistent with experiential-dynamic trial-therapy maps and affect-phobia pedagogy (13–18, 25). Eye-contact trial → fogging/tunnel vision → C → stop deepening; regulate → endurance: three grounding cycles/session; re-check 8–12 weeks. Protection of a positive state after progress appears in Section 5.2, Episode 2.

### 3.4 Protecting positive affect when punitive dynamics activate

Because punitive dynamics often strike precisely at moments of progress, the therapist anticipates the pattern and intervenes in four moves. First, name the switch from a positive affect to an attack. Second, externalize the persecutory voice so it can be addressed without confusing it with the self. Third, add compassion and limits so that the adaptive state can be held for a short interval without punishment. Fourth, link the cost of complying with the attack to concrete functional losses such as diminished endurance, disrupted planning, or unstable dyadic relatedness (8, 10, 11, 16–18). In robust structures, brief confrontation of the punitive pattern may follow protection; in fragile structures, confrontation is minimal and delayed. The aim is consistent: preserve and consolidate nascent adaptive states before any renewed deepening. This protects alliance, widens the working window over time, and is compatible with an active-inference account in which positive states are newly inferred priors that require precision support to persist (15–20). A worked documentation line is illustrated throughout Section 5.2 and consolidated in Episode 6.

### 3.5 Action and the documentation sentence

Every micro-cycle closes with one disciplined sentence that records what happened and translates the choice into functional language for team use. The template uses five fields in words: the triggering cue, the observable response, the threshold appraisal, the action taken, and the Mini-ICF-APP impact and re-check interval. Re-check intervals refer to review points for functional targets (Mini-ICF-APP domains) and can align with routine team reviews; they do not imply a specific psychotherapy session frequency. For example, a direct focus on a conflict with a manager is followed by breath-holding and an upper-abdominal cramp and is judged as a drift within the working range toward collapse; the action is downregulation, partial blocking, and a 2-second eye-contact exposure; the expected impact is lower endurance and oscillating dyadic relatedness under attachment load, with a re-check in 6 to 8 weeks. Sharing a success is followed by a rapid shift from joy to self-attack and collapse, judged as collapse by shame; the action is protection of the positive state, externalization of the critic, and compassionate limits; the expected impact is improved assertiveness when positives are protected for 20 to 30 seconds, with one-weekly practice of sharing a positive event and a re-check in 4 to 6 weeks. A closeness probe is followed by joking and idealization within the working window; the action is clarification of the detour, a return to feeling, and rehearsal of one concrete request; the expected impact is improved planning/structuring and assertiveness with a 6-week re-check. Because fields are standardized-node, threshold, pathway, action, and functional target, the sentence justifies the intervention from observable data, ties it to Mini-ICF-APP domains for interprofessional communication, and accumulates episode-level labels suitable for supervision and, where consented, later research on reliability or decision support (7, 8, 10, 11). Copy-ready templates and pseudocode live are shown in **Supplementary Material S1**. Ask rehearsal → joking detour → A–B → clarify/block; one sentence to feeling → assertiveness: one graded request/week; re-check 6 weeks. Direct phrasing examples for endurance, planning, assertiveness, and dyadic relatedness are rehearsed in Section 5.2, Episodes 1–4.

### 3.6 The Mini-ICF-APP bridge in prose

CSA outputs are rendered in the shared language of functioning because teams coordinate care, justify intensity, and evaluate recovery in those terms. Instead of tables, concise phrases are appended to the documentation sentence. Endurance and persistence can be described as declining under attachment-laden triggers near the boundary to collapse, with a plan to titrate closeness in 5- to 10-second windows and reassess capacity in 6 to 8 weeks. Decision making narrows as anxiety constricts the field; after regulation and partial blocking, restoration of simple two-step decisions is expected with reassessment in 6 weeks. Planning and structuring degrade when detours proliferate under closeness; a checklist scaffold with one request rehearsal per week is appropriate, with reassessment in 6 to 8 weeks. Rule adherence and social participation may destabilize under humiliation cues; with a rehearsed safety script inside the working window, steadier group participation is anticipated and reviewed in 4 to 6 weeks. Assertiveness and dyadic relatedness rise when positive states are protected, allowing graded requests and more stable contact over 4 to 6 weeks (6–8, 10, 11). These phrases follow published validation and implementation work on the Mini-ICF-APP and its short forms, ensuring that the same micro-observations that guide safe dosing also justify stepped-care intensity and set team-ready targets and timelines (see **Supplementary Material S3** for phrase bank) (6–8, 10, 11). This closes the loop from structure to severity to function, while keeping language interoperable across disciplines and settings. Worked illustration (integrative paragraph): a therapist asks about a conflict with a manager. The patient replies that it is fine and that they simply work harder, while their shoulders tighten and breathing shallows. Defense (isolation of affect) is the front of the system with striated-pathway anxiety and a working-range threshold. A graded dose is chosen: a partial block of the detour, a brief embodied check-in, and a two-sentence focus on the wish. The patient then reports a stomach flip, indicating smooth-muscle activation and proximity to collapse, so the therapist pauses to co-regulate. The patient tolerates a 2-second eye-contact step (progression), followed by a harsh inner verdict with head drop (superego/shame). The therapist names the switch, externalizes the punitive voice, adds compassion and limits, and protects the positive state for 20 to 30 seconds before any further deepening. The documentation sentence links these observations to functioning—anticipating reduced endurance and unstable dyadic relatedness under dyadic demand—and sets review intervals that the team can use (6–8, 10, 11, 13–18). Training, supervision, and interoperability. Because the CSA reduces work to observable signals, thresholds, and short prompts, it is highly trainable. Verbatim prompts and micro-dose timing can be practiced in brief drills; psychodiagnostic guardrails—resistance level, anxiety pathway, character versus tactical defenses—keep dosing aligned with tolerance; and the one-line documentation standardizes quality review across teams. This is consistent with emerging evidence for deliberate practice and structured feedback in psychotherapy training and with the feasibility of human-in-the-loop computational assistance that flags alliance-relevant or threshold-transition cues from session language and paralinguistics without dictating care (29, 30, 32, 35, 36, 42). Because each episode sentence already encodes severity and function, the mapping to ICD-11 and AMPD, and Mini-ICF-APP is now made explicit. *Bridge*. Having specified the bedside routine, we now show how its outputs travel across nosology and team documentation.

### 3.7 Minimal machine-readable schema and state diagram

**Figure 1:**
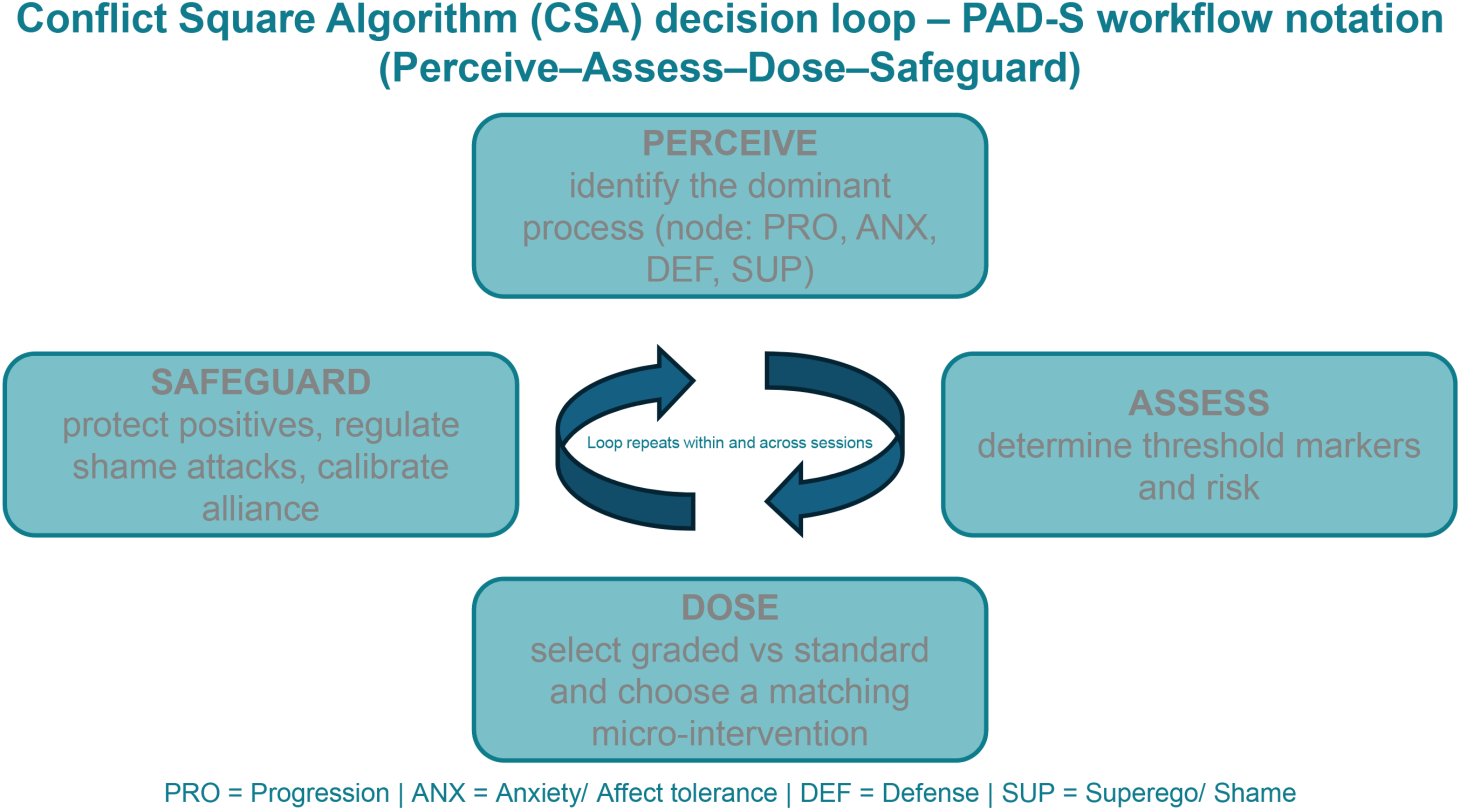
The Conflict Square Algorithm (CSA). The algorithm is applied within a continuously repeating loop. Step 1 estimates the current safety threshold (A–C) based on protective strategies, emotional arousal/tolerance, and risk markers. Step 2 identifies the currently dominant node among protective strategies (P), emotional arousal and tolerance (A), constructive movement toward goals (D), and self‑attacking/shaming processes (S). Step 3 selects node‑appropriate interventions and doses them according to threshold A–C. Step 4 records impact on functioning, safety, and engagement, informing the next iteration.

**Figure 2:**
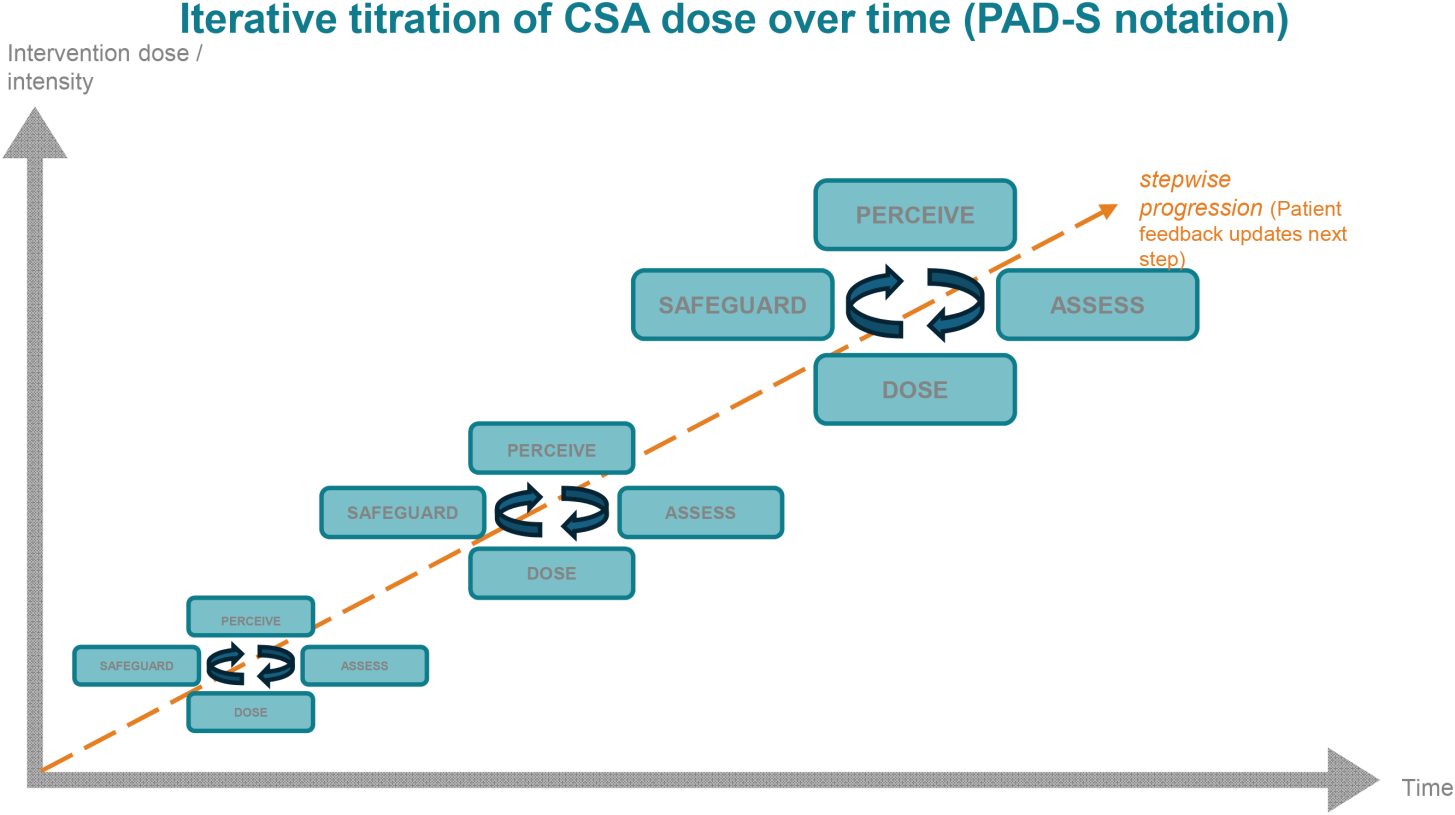
Iterative and incremental application of the PAD-S cycle across time and intervention dose. Across sessions (x-axis), the same P-A-D-S loop is applied repeatedly while intervention intensity/dose (y-axis) is increased in stepwise increments as tolerance and stability improve. Early in fragile states, the clinician uses small cycles with frequent safeguard steps and strict stop rules; as the workable window widens and post-progress punitive attacks decrease, cycles can be extended, and the dose can be increased without crossing thresholds. PAD−S, Perceive–Assess–Dose–Safeguard.

To support reproducibility and future human-in-the-loop implementations, CSA episode outputs are defined as a small set of required fields that can be stored in notes, registries, or research datasets. The schema below is intentionally minimal and human-readable; it does not imply automation of care. In companion work, the same core logic is presented as the PAD-S decision matrix; the fields map one-to-one to the CSA episode line used here (21). **Figure 1** summarizes the PAD-S micro-decision loop, and **Figure 2** illustrates its iterative and incremental application across time and intervention dose.

Note on the “perceive” step. In PAD-S terminology, perception is not limited to semantic content. Clinicians integrate verbal content with paraverbal prosody and non−verbal behavior (e.g., gaze, posture, and tempo) and treat the patient’s immediate response to each micro−intervention as the primary feedback signal for calibration. Because complete decentering is impossible for human clinicians, structured supervision and optional human−in−the−loop feedback tools (e.g., audio−derived prosody markers and optional heart−rate biofeedback) can support calibration—without shifting decision authority away from the clinician.

Pseudo-code summary (high level):

for each clinically meaningful moment:

node = perceive(front_of_system) # DEF | ANX | PRO | SUP
thr = assess_threshold(pathway_markers) # A–B | B→C | C
act = choose_dose(node, thr) # standard vs. graded + stop rules
safeguard(positives, shame_attack, rupture) # protect positives; regulate; repair alliance
log_episode(trigger, observables, node, thr, act, icf_target, recheck_interval)

Machine-readable episode schema (JSON example):

{
“patient_id”: “ID-XXX”,
“date_time”: “YYYY-MM-DDThh:mm:ss”,
“setting”: “outpatient”,
“trigger”: “Explore closeness with partner”,
“observable_response”: “Breath-hold + upper-GI cramp; global self-attack after pride”,
“node”: “ANX -> PRO -> SUP”,
“threshold”: “B -> C”,
“action”: “Down-regulate; protect positives; 2-s graded exposure”,
“icf_impact”: “Endurance and dyadic relatedness narrow under attachment load; recheck 6-8w”,
“icd11_severity_sentence”: “PD - severity: moderate”,
“structural_note”: “Mixed structure; smooth-muscle B with B->C drift after progress”,
“stop_rules_invoked”: [“Halt deepening at C-signs”, “Protect positive affect”]
}

Recommended registry fields and quality assurance procedures (e.g., clip-based calibration for A/B/C and double-coding 5%–10% with kappa targets) are provided in the **Supplementary Material** to keep the main text concise.

## 4 Mapping to nosology and functioning

This section anchors moment-to-moment clinical observations in the dimensional language of contemporary nosology and translates them into team-ready statements of functioning. In prose, we link the CSA—four observable decision nodes (defense, anxiety/affect tolerance, progression, and superego/shame) gated by A–C safety thresholds—to the severity-first stance of ICD-11 and to the impairment-first architecture of the DSM-5 Alternative Model for Personality Disorders (AMPD). We then show how a single in-session “episode line” can be rendered directly in Mini-ICF-APP terms for care planning, stepped-care decisions, and administrative or forensic communication. The full crosswalk and phrase bank are available in **Supplementary Material S3**.

### 4.1 Why severity first: locating CSA inside ICD-11

ICD-11’s graded approach to personality disorders and other conditions makes functional impairment and severity the primary axes for clinical communication and service planning. In practice, the same category can contain patients with very different capacities under interpersonal load; the CSA adds the bedside piece by eliciting how the person performs as relational demand rises and by expressing that performance as a brief severity-plus-safety sentence (for example, “mixed structure with narrowing window under attachment, superego attacks after small gains; graded format indicated”). This aligns with scholarship detailing the ICD-11 pivot to severity and the growing empirical base for severity measures, including the PDS-ICD-11 and its reliability and validity across samples. The conceptual and measurement bridge here is supported by work on ICD-11 implementation and by empirical studies of structural integration and functioning (3, 22, 23, 43–47).

### 4.2 Convergence with the AMPD: impairment first, traits second

The AMPD separates the level of personality functioning (self-direction and interpersonal functioning) from maladaptive trait configurations. CSA maps naturally to this structure: defense patterns and superego/shame dynamics inform self-regard and self-direction, progression indexes agency in real time, and anxiety/affect tolerance constrains relatedness and participation when load increases. In other words, the very signals that guide CSA dosing and safety correspond to the AMPD impairment axis, while trait information remains useful for risk and relapse formulations. This conceptual fit allows a CSA sentence to travel alongside an AMPD impairment rating without duplication of effort (3, 4, 45, 47, 48).

### 4.3 From an “episode line” to Mini-ICF-APP: function as shared currency

Each CSA microcycle is closed with one disciplined sentence trigger, observable response, threshold, action, and functional impact with a re-check interval. Expressing the last field in Mini-ICF-APP terms (endurance/persistence, planning/structuring, decision making, assertiveness, dyadic relatedness, group interaction, and rule adherence) makes the observation interoperable across disciplines (see **Supplementary Material S3** for phrase bank). Validation and utility studies of the Mini-ICF-APP and its derivatives demonstrate psychometric adequacy and practical value in routine care, inpatient programs, and forensic or work-capacity settings, supporting its use as the lingua franca for goals and audits (6–11). Two brief illustrations show the tone and specificity without recourse to diagrams. First, in a mixed structure with narrowing tolerance under attachment, one anticipates temporary dips in endurance and planning/structuring and oscillations in dyadic relatedness near the boundary between workable and non-workable arousal. Targets may include practicing micro-closeness in 5- to 10-second windows and rehearsing one graded request per week, with review at 6 to 8 weeks. Second, in severe fragility with signs of cognitive-perceptual disruption at minimal load, one predicts sharp reductions in endurance and group interaction; the action is to protect positive states explicitly, limit exposure to seconds, and institute a team-wide shame-repair script with review at 8 to 12 weeks (6–8, 10, 11).

### 4.4 What changes across structural bands: three prose vignettes

High resistance (neurotic organization). Symbolization remains intact, and anxiety discharges in striated musculature within the workable window; progression appears as clear wishes and small approach steps. A standard format—naming and blocking tactical defenses, and then deepening the affect in short bouts—is justified. Mini-ICF-APP targets often include assertiveness and decision making with stable rule adherence (13, 16–18, 25). Mixed structure (narrowing window under attachment). As proximity increases, anxiety shifts from striated to smooth muscle, the workable window narrows, tactical detours reappear, and brief superego micro-attacks may follow small gains. Graded work—partial blocking, early regulation, and seconds-long exposures—becomes the safer default. Functional phrasing anticipates transient decreases in endurance and planning/structuring and fluctuating dyadic relatedness that are converted into time-bounded goals with explicit re-checks (8, 13, 16–18, 25). Severe fragility (disruption at minimal load). Primitive defenses emerge early; cognitive-perceptual signs and persecutory superego processes appear with little provocation; positive states tend to flip into collapse. Graded work with strict stop rules and protection of positive affect before any deepening is indicated, with functional planning focused on endurance, group interaction, and decision making under minimal, controlled demand (8, 13, 16–18, 25).

### 4.5 How the mapping supports stepped care, level of care, and forensic decisions

Because a CSA episode already couples severity with function, it supports stepped-care logic: step up intensity when the workable window narrows and non-workable events recur; step down when the window widens and Mini-ICF-APP targets improve on schedule. Service models that reduce fragmentation and coercion, such as track-based care that bridges sectors and recovery-oriented inpatient models, are natural habitats for this documentation style because it clarifies triggers, safety scripts, and prerequisite skills for transition between levels of care. The evidence base for track treatment and for recovery-oriented redesigns that reduce coercive measures illustrates the systems-level payoffs of making severity and function transparent and auditable (33, 34).

### 4.6 Why thresholds matter for the bridge from nosology to function

The A–C thresholds are integral to safety and dosing. They specify when work remains therapeutic and when risk outweighs benefit. Observable markers—shifts across striated, smooth-muscle, and cognitive-perceptual pathways; posture and prosody during shame; and lexical signs of global self-condemnation—make thresholding reproducible across raters and settings. Embedding these thresholds in the nosology-plus-function sentence ensures that “severity” is not just a label but a capacity profile tied to concrete actions and timelines (for example, graded micro-exposure and protection of positive states, with defined re-checks). This is why we recommend appending a compact CSA line to ICD-11 codes in summaries and discharge letters (8, 10, 11, 22, 23, 45, 47).

### 4.7 Compatibility with computational psychiatry—without dictating care

The CSA creates clean labels—node, threshold, and action—that are suitable for consented, human-in-the-loop computational pipelines. Session language and paralinguistic signals can, in principle, flag boundary crossings or post-progress superego activation for supervision and fidelity feedback without dictating clinical choices. Recent work demonstrates that alliance-relevant linguistic features can be detected, and programmatic efforts map alliance dynamics from session language; theoretical frameworks in active inference provide a principled account of therapy as iterated perception–action cycles in which small, safe prediction errors support learning. In this view, thresholds stabilize learning by preventing catastrophic updates, and protection of positive states preserves precision on newly adaptive priors (19, 20, 27–30, 39).

### 4.8 Limits and where the details live

The present mapping is conceptual and operational rather than a validated diagnostic instrument. Reliability studies on node/threshold agreement and outcome studies on safety, alliance, and functional gains are planned and are feasible given the existing psychometric base for severity measures and functioning tools. Rules, prompts, and stop criteria are available in **Supplementary Material S1**, annotated transcripts in **Supplementary Material S2**, and the Mini-ICF-APP crosswalk and phrase bank in **Supplementary Material S3** (6, 8, 10, 11, 22, 47). Take-home ICD-11’s severity-first pivot invites a shift from label to capacity. The CSA supplies a minimal, teachable way to elicit those capacities *in vivo*—four nodes and explicit thresholds—and to render them as a brief severity-plus-safety-plus-function sentence that aligns clinical micro-decisions with the Mini-ICF-APP and with the AMPD’s impairment axis. The crosswalk is ready for teams; the prose here keeps the method legible while pointing to supplements for copy-ready phrasing (3, 4, 6, 8, 10, 11). Bridge. The following case knits the rules into a single narrative and anchors each step in lived interaction. We now thread these rules into a single composite case, keeping node, threshold, and action visible sentence by sentence.

## 5 Clinical case illustration

Ethical note. The case was a composite assembled from routine care to preserve privacy. Identifying details were altered. Interventions followed the CSA in a manner consistent with contemporary experiential-dynamic pedagogy; short, time-stamped excerpts used for rater training are available in **Supplementary Material S2**.

### 5.1 Setting and baseline (severity, type, and risks)

Case map (Ms. K.)—Window: A–B workable; B → C under attachment; C at minimal load under stress. Typical pattern: PRO → SUP rapid reversal after small gains. Action: protect positives before any renewed deepening. Mini-ICF-APP forecast: endurance↓ and dyadic relatedness oscillations near B → C; graded requests weekly; re-check 6–8 weeks. “Ms. K.” was a 34-year-old nurse on partial sick leave after recurrent interpersonal ruptures at work. She reported episodic derealization and a wave of self-disgust that followed moments of pride or appreciation. History included chronic humiliation in a caregiving relationship and recent negative feedback from a supervisor. Baseline nosology was ICD-11 personality disorder, moderate severity, with unstable self-concept, hypersensitivity to social threat, and cognitive-perceptual destabilization when attachment load rises; persistent depressive disorder (mild) and social anxiety were comorbid (3, 4, 22, 23, 47). Functional assessment with the Mini-ICF-APP indicated constraints in endurance or persistence, planning or structuring, assertiveness, and dyadic relatedness; these domains served as shared targets and re-check intervals for the team (6–11). Because destabilization tends to occur under small interpersonal demands, dose selection prioritized safety and graded pacing. We translated each clinically meaningful moment into a single sentence that can be reused across disciplines in Mini-ICF-APP language. A brief psychodiagnostic sample showed anxiety initially in striated musculature (thoracic tightening and breath-holding), with rapid migration to smooth-muscle phenomena (upper-abdominal “flip”) as interpersonal closeness increased; at brief peaks, cognitive-perceptual disruption appeared (fogging and tunnel vision). Defenses oscillated between tactical detours (joking and topic shifting) and syntonic self-accusation. Superego or shame markers frequently followed micro-progressions: gaze drop, posture collapse, and global condemnation like “I am ridiculous”. This mix indicated graded work from the outset, with explicit safety thresholds, stop rules at cognitive-perceptual signs, and protection of positive affect before any confrontation. These parameters followed established ISTDP scaffolds used to gate standard versus graded formats and to time defense work relative to tolerance (13, 14, 16–18, 25).

### 5.2 Episode line in prose (trigger, markers, threshold, dose, and Mini-ICF target)

Episode 1—Attachment-laden inquiry. Early in Session 1, the therapist invited a brief review of a recent praise event at work. Ms. K. showed immediate striated tension (clenched hands and held breath) and a thin, joking detour. Symbolization remained intact but narrowed: this is the working range. The therapist chose a graded move: partial blocking of the detour, breath-paced co-regulation, and an invitation to name the affect in two short sentences, followed by a pause to check arousal. The documentation sentence closed with a functional link: begun micro-assertiveness in brief appreciation exchanges and re-checked within 6 to 8 weeks (6, 8, 10, 11). Episode 2–Progression followed by superego attack. After brief regulation, Ms. K. voiced a wish (“I wanted to feel proud”), tolerated eye contact for a few seconds, and then dropped her gaze with a global verdict (“Pride is pathetic”). The sequence—brief brightening followed by a harsh inner verdict and partial collapse—signaled proximity to the upper boundary of the workable window. The therapist named the switch, externalized the punitive voice, affirmed compassionate limits (“we will not punish a good moment”), and rehearsed seconds-long exposure to pride with immediate buffering. The documentation sentence encoded the safety choice and functional translation: protect the positive state first, set one protected appreciation exchange per week, and re-check in 4 to 6 weeks (16–18). Episode 3—Closeness and smooth-muscle migration. When the therapist proposed a behavioral micro-enactment (“What words might you use to ask a colleague for help?”), Ms. K. named the request but clutched her abdomen and shortened her speech. This was still workable but drifting toward collapse. The therapist downshifted with paced exhalation and orienting, partially blocked the return to logistics, and re-linked the wish to action in two-sentence cycles. The functional forecast noted that planning and structuring often degraded under attachment-laden tasks; a three-step checklist and one *in vivo* graded ask per week were set with a 6- to 8-week review (6–8, 10, 11). Episode 4—Tactical defense and process strain. Asked about the partner’s response to closeness, Ms. K. joked and globalized with icy compliance. Symbolization is intact; defense is foremost. Within a graded frame, the therapist named and cost-linked the detour, invited one sentence of feeling toward the partner, and capped exposure at 10 to 15 seconds with a regulation check. The action was tied to assertiveness at home—a single value-congruent “I-statement” weekly—while anticipating superego micro-attacks after any success and pre-emptively protecting the positive state (13, 14, 16–18). *Episode 5—Cognitive-perceptual stop rule*. When asked to imagine making the request that evening, Ms. K. showed fogging, a narrowed gaze, and fragmenting speech. Deepening stopped. The therapist grounded, re-oriented, restored the working window, and postponed affect work until symbolization returned. The functional note anticipates transient reductions in endurance and group interaction during such events and installs a brief safety routine of three grounding cycles per session; re-check after 8 to 12 weeks (6, 8, 10, 16–18). Episode 6—Consolidation and rehearsal. With the working window restored, the therapist validated a clear request for the next team meeting, briefly amplified the progression, rehearsed the words, and anticipated post-success shame with an explicit protection step lasting roughly 0.5 minutes. The documentation sentence linked the micro-gain to dyadic relatedness at work with one graded request per week and a 4- to 6-week review (6, 10, 11, 16–18).

### 5.3 Progression and superego “rapid reversal after a positive moment” (protecting the positive)

Across the first three sessions, each forward step—naming a wish, tolerating a few seconds of eye contact, and accepting praise—was followed quickly by punitive self-talk and partial collapse. The working rule is to protect positive states before any renewed deepening: name the switch, externalize the critic, pair compassion with limits, and resume exposure only within the demonstrated working window. This sequence prevents overshoots into cognitive-perceptual disruption, preserves alliance, and allows adaptive micro-states to consolidate long enough to become reference experiences. Contemporary ISTDP teaching and deliberate-practice pedagogy emphasize the same order of operations: track the leading process, gauge threshold, buffer positive affect, and then time confrontation relative to tolerance (13, 14, 16–18, 36). From a computational perspective, these micro-cycles can be viewed as small, safe perturbations that support learning within an iterative perception–action loop. Protection of positive states stabilizes newly adaptive predictions about closeness, graded exposure near the upper boundary increases information gain without catastrophic update, and externalizing the punitive voice reduces the weight assigned to harsh priors. This interpretive bridge does not dictate care but clarifies why brief, titrated steps and explicit stop rules enhance stability (19, 20).

### 5.4 Brief follow-up on functional goals

Over 6-weekly sessions (one missed for scheduling), no adverse events occurred; two imminent cognitive-perceptual episodes were managed promptly with stop rules and grounding. Alliance ratings improved from “uncertain” to “hopeful”. A trainee and supervisor coded episode lines in parallel, showing consistent use of the CSA sentence format, which supports teachability and inter-rater discussion in supervision (36). Functional phrasing, kept in the style used by interprofessional teams, read as follows. Endurance or persistence improved from markedly reduced under dyadic demand to mildly reduced, with three daily grounding cycles; the review interval was shortened from 8–12 to 6–8 weeks. Planning or structuring improved through a three-step checklist for attachment-laden tasks; transient dips under humiliation cues recovered after regulation; next review in 6 weeks. Assertiveness increased to two graded requests at work and one value-congruent statement at home per week; positive states were explicitly protected for 20 to 30 seconds to avoid post-success collapse. Dyadic relatedness still oscillated near the upper boundary of the working window, but 10- to 15-second micro-doses were now tolerated without collapse; the next step was one protected appreciation exchange per week (6–11). Case takeaway. Identify what is foremost in the moment among defense, anxiety, or affect tolerance, progression, and superego or shame; gate intensity by explicit thresholds and treat cognitive-perceptual signs as a stop condition; protect positive affect first when punitive dynamics strike after progress; and encode each micro-decision as a compact sentence that already states the expected functional impact and the review interval. This routine is teachable, auditable, and compatible with ICD-11 severity and Mini-ICF-APP documentation in stepped-care services (3, 4, 6, 8, 10, 13, 14, 16–18, 22). The same fields that structure the case (node, threshold, action, and Mini-ICF) support scope limits, training, and reliability designs detailed next.

### 5.5 Worked examples (consecutive micro-episodes)

Worked micro‑episode examples are summarized in **Tables 2**–**4**.

**Table 2:** Worked micro-episodes: Example 1 (robust to mixed structure; standard format with titration).

Episode	Trigger/probe	Leading node	Threshold	Markers (examples)	Dose + next action
1	Conflict with supervisor (specific example)	DEFENSE	A → B	Coherent narrative; mild hand clenching	Standard: clarify detour; invite feeling specificity.
2	Invite feeling toward supervisor	ANXIETY	B	Sighing respiration; shoulder tension	Briefly regulate; keep focus short (10–20 s).
3	Return to feeling + wish	PROGRESSION	B	Names anger + wish; maintains contact	Standard: deepen 1 step; ask for bodily signal.
4	Patient jokes/intellectualizes	DEFENSE	B	Humor detour; eye contact avoids	Partial block; return to task; keep dose moderate.
W5	Direct statement of boundary (role-play)	PROGRESSION	B → A	Agency increases; anxiety reduces	Rehearse 1 concrete ask/week; document Mini-ICF target (assertiveness).
6	Mild guilt without collapse	SUPEREGO (guilt)	B	Self-criticism but stable posture	Differentiate guilt *vs*. shame; compassion + limits; resume work after stabilization.

**Table 3:** Worked micro-episodes: Example 2 (smooth-muscle B with B → C risk and post-progress shame reversal).

Episode	Trigger/probe	Leading node	Threshold	Markers (examples)	Dose + next action
1	Closeness probe (seconds-long)	ANXIETY	B	Breath-hold + upper-GI cramp (smooth muscle)	Graded: downregulate; bracing; 2-s exposure only.
2	Return to narrative detour	DEFENSE	B	Topic shift; joking	Partial block; clarify cost; keep pace slow.
3	2-s eye contact step	PROGRESSION	B	Maintains contact briefly	Reinforce; repeat once; stop before drift.
4	Pride appears and then self-attack	SUPEREGO (shame)	C	Head drop; global verdict; voice fades	Stop deepening; protect positive affect; externalize critic; restore B.
5	Grounding and re-orientation	ANXIETY regulation	B → A	Cognitive clarity returns	Regulate; return to safe task.
6	Micro-request rehearsal	PROGRESSION	A → B	Stable contact; mild striated anxiety	Graded rehearsal; set daily 5-s practice; document endurance/relatedness target.

**Table 4:** Worked micro-episodes: Example 3 (fragile structure with CPD; C-level stop rules).

Episode	Trigger/probe	Leading node	Threshold	Markers (examples)	Dose + next action
1	Minimal affect focus	ANXIETY	C	Fogging; losing track of thoughts; tunnel vision risk	Immediate stop; orient; reduce relational load.
2	Restore clarity (here-and-now)	ANXIETY regulation	B → A	Attention stabilizes	Grounding; pacing; check medical/medication confounds.
3	Low-load task cue	DEFENSE	A	Vagueness without anxiety signaling	Invite specificity; gentle clarification.
4	Seconds-long exposure to feeling word	PROGRESSION	B	Brief contact with feeling; no CPD	End exposure early; reinforce; schedule micro-practice.
5	Monitor for punitive swing	SUPEREGO	B	Self-attack signs begin	Protect positives; limit critic; keep work shallow.
6	Return to stabilization	ANXIETY regulation	A	Stable breathing; coherent speech	Close loop; document stop rules invoked and re-check interval.

CPD, cognitive-perceptual disruption.

Aim: Demonstrate transition logic and dose modulation over consecutive moments. All examples are condensed, de-identified composites designed for didactic illustration and rater calibration.

## 6 Clinical scope and usage

### 6.1 Primary positioning

The CSA is first a scaffold for training, supervision, and quality assurance. It standardizes moment-to-moment observation and decision making using four observable nodes—defense, anxiety and affect tolerance, progression, and superego or shame—gated by three thresholds of the window of tolerance. Each decision ends with a one-sentence record that can be read by teams in the language of functioning. This emphasis on teachable micro-skills and structured feedback aligns with contemporary pedagogy on deliberate practice and with experiential-dynamic psychodiagnostics that link pathway markers and thresholds to dosing rules. The result is a compact routine that clinicians can rehearse on video and then apply judiciously *in vivo* (16–18, 25, 35, 36). When live, in-session use is appropriate. The CSA can be applied directly in sessions when three guardrails are in place: the clinician can identify which node is leading at this moment; the immediate threshold is clear-regulated, narrowing but workable, or cognitive-perceptual disruption or shame collapse; and the next action can be titrated safely, including immediate protection of positive affect when punitive processes strike after a forward step. These conditions mirror established decision trees in intensive short-term dynamic work, where graded formats and strict stop rules are used to prevent overshoot into destabilization (14, 16–18). Target populations and settings. Because the CSA codes generic, observable features—defensive maneuvers, pathway shifts, small approach steps, and punitive attacks—it travels across orientations and levels of care. It is especially useful in ambulatory and integrated psychosomatic services for mood and anxiety disorders, trauma-related difficulties, somatic symptom presentations, and personality-spectrum problems where tolerance and alliance fluctuate. Evidence syntheses in intensive short-term dynamic psychotherapy, including cost and service outcomes, support the feasibility of dosing by pathway and capacity; affect-phobia manuals provide convergent instruction on graduated exposure with regulation (12–18, 25, 37, 38). Contraindications and cautions (**Table 5**). The algorithm should not be used to push deepening when baseline signs of cognitive-perceptual disruption are present, or when the alliance cannot sustain even a few seconds of exposure. In such contexts, it remains a supervisory compass and documentation scaffold while clinical work emphasizes co-regulation, alliance repair, environmental supports, and practical scaffolding. Surface calm must not be equated with high tolerance: some configurations show low observable anxiety and marked intolerance to closeness, pride, or grief. In these cases, proximity is graded, limits are explicit, and positive states are protected rather than confronted. This distinction—structure and defenses versus process resistance in the room—prevents mis-dosing (16–18, 25). Standard and graded formats in practice. Standard format is suitable when the working window is stable and symbolization is intact. Graded format is indicated when fragility or mixed structural features narrow the window or when the trend moves toward collapse. Details are specified in Section 3.3. Choosing the next action and documenting it. To keep choices auditable and interoperable, each micro-cycle closes with one sentence in words: the clinically meaningful trigger, the observable response, the threshold just judged, the action selected, and the expected impact stated in Mini-ICF-APP terms with a review interval. Because the fields—node, threshold, pathway, action, and target—are standardized, teams can read the note, supervisors can rate fidelity, and services can aggregate episode-level signals without changing local workflows. Validation studies of the Mini-ICF-APP and its short forms support this bridge from process to functioning (6, 8, 10, 11). Safety architecture. The CSA’s core safety features are explicit thresholding by physiology and behavior, mandatory stop rules when cognitive-perceptual signs or shame collapses appear, and active protection of positive affect before any renewed deepening. These practices are designed to minimize over-exposure, preserve alliance, and consolidate adaptive states; they reflect widely taught safeguards in experiential-dynamic work with fragile structures (14, 16–18, 25). Mapping to nosology and function in routine care. The algorithm supplements the severity-first stance in contemporary nosology by rendering bedside capacity profiles visible—window width, pathway shifts, and risk of punitive attack—and then translating them into the language of functioning used in interdisciplinary plans. That is why two people with the same category can justifiably receive different intensity, pacing, and safeguards. The episode sentence travels from therapy notes to team goals, providing a practical link between categorical diagnosis, dimensional severity, and functional recovery. Compatibility with the Alternative Model for Personality Disorders follows from the shared focus on impairment in self and interpersonal functioning (3, 4, 22, 23, 45, 47). Service fit and stepped care. Because episodes already encode severity and function, they support transparent triage and level-of-care decisions: step up when the workable window remains narrow and destabilization recurs; step down when the window widens and functional targets are met on schedule. Track-based and recovery-oriented models illustrate how structured language and time-boxed targets can reduce coercion and improve continuity across sectors; the CSA helps generate those justifications at the point of care (33, 34). *Bridge*. With the scope defined, we outline a pragmatic validation program and options for human-in-the-loop assist. Digital augmentation (human-in-the-loop). Standardized episode lines create clean labels for computational psychiatry without dictating care. With consent and governance, language and paralinguistic markers can flag impending boundary crossings or post-progress punitive attacks for supervision and fidelity feedback, while clinicians remain in control of dosing and timing. This advisory, not prescriptive posture fits active-inference accounts of therapy as iterated perception–action under uncertainty: small, safe prediction errors drive learning, and explicit thresholds stabilize the loop. Early demonstrations of alliance mapping from session language and protocolized fidelity feedback show the promise of assistive tools when they are transparent and embedded in workflow (19, 20, 27–30, 32). Training and quality recommendations. Introduce the CSA through short, video-based drills that ask trainees to label node, threshold, dose, and functional target on brief clips; provide structured feedback; and run periodic reliability checks on node and threshold calls. This approach is consistent with evidence on deliberate practice for interpersonal skills and with the validation literature on functioning measures used for documentation and audit (8, 10, 35, 36). Summary stance. In supervision and training, the CSA helps clinicians see what is leading now, choose the next safe step, and state a functional expectation that teams can follow. In live encounters, use it when thresholds and alliance can be actively maintained; otherwise, keep it as a supervisory compass rather than a real-time coding grid. The method is intentionally modest: a concise grammar that links micro-process to function, interoperable with dimensional nosology and ready for measured, human-guided computational support (3, 4, 6, 8, 10–18, 22, 25, 30, 37, 38, 45, 47).

**Table 5:** Scope of applicability and contraindications (rapid checklist).

Use CSA in-session when …	Do NOT use CSA as a real-time dosing tool when …
- The setting allows slowing/stopping without time pressure. - Clinician can track somatic and cognitive-perceptual markers of anxiety tolerance. - Basic alliance is present or can be repaired with low-load moves. - Patient is oriented and not intoxicated; language contact is sufficient for in-session micro-testing. - CSA is used as a scaffold for safety, documentation, and team communication (not as automation).	- Acute safety risk dominates the encounter (imminent self-harm/violence, severe intoxication, and delirium). Follow local emergency/risk protocols first. - Severe cognitive impairment or active medical instability makes symptom interpretation unreliable. - High suicidal ideation is present, and the encounter cannot maintain safety; ISTDP trial-therapy literature treats suicidality as a contraindication for deepening formats (41). - The clinician cannot reliably detect CPD/shame-collapse markers or cannot pause/repair when C-level signs appear. - The context incentivizes premature intensification (e.g., coercive/unstable setting) rather than graded titration.

CPD, cognitive-perceptual disruption.

### 6.2 Implementation shortcut

If unsure, use CSA primarily as a supervisory and documentation compass (episode lines), and restrict in-session application to low-load probes with explicit stop rules.

## 7 Empirical validation and computational bridges

### 7.1 Aim

To transition the CSA from a conceptual teaching scaffold to a reliable, auditable, and assistive instrument, this section outlines a staged program spanning inter-rater reliability, treatment integrity, patient-level outcomes, and human-in-the-loop (HITL) computational augmentation. Throughout, clinical primacy is retained: the CSA remains a therapist-facing decision aid; any digital component is advisory, transparent, and auditable rather than a black box. The basic analytic unit is the documented micro-episode, rendered in words as a trigger to observable response to threshold (A, B, or C) to action to functional impact (Mini-ICF-APP). Evidence and precedents from ISTDP, active inference, and contemporary training science inform the design and measurement strategy (12, 13, 26, 35–39).

### 7.2 Feasibility demonstration (proof-of-concept annotation)

Aggregate coding counts for the three training‑video demonstrations are summarized in **Table 6**.

**Table 6:** Feasibility demonstration (proof-of-concept): aggregate speaker-turn coding counts across three published ISTDP training videos (N = 2,809).

Transcript	N turns	DEF Pt)	PRO (Pt)	ANX (Pt)	Invite PRO (Th)	DEF work (Th)	ANX reg. (Th)
Restructuring Projection in a Borderline Patient	1,087	87	405	54	316	159	66
Treatment of the Fragile Patient	345	43	75	54	92	26	55
Treatment Resistance: The Addict who had “no problem”	1,377	577	89	23	399	278	11

Limitations: thresholds (A–C) were not coded in this feasibility pass; therefore, this table does not address inter-rater agreement for threshold calls. The next step is a two-rater calibration study on short clip sets, followed by double-coding of 5%–10% of sessions to estimate kappa for node and threshold identification.

To provide an initial feasibility signal (not a reliability study), we transcribed and single-rater coded three published psychotherapy training videos (Treatment Resistance: The Addict who had “no problem”; Restructuring Projection in a Borderline Patient: Instructional Video Bundle; and Treatment of the Fragile Patient) distributed by the ISTDP Institute at the speaker-turn level (therapist and patient) (49–51). We report only aggregate counts and do not reproduce any transcript excerpts. The aim is to show that the coarse-grained node mapping can be applied at scale and produces analyzable labels.

Corpus size: N = 2,809 speaker turns across three video-derived transcripts.

### 7.3 Reliability and treatment integrity

*Targets and materials*. Reliability concentrates on two readouts: the front of the system node (defense, anxiety, or affect tolerance, progression, superego, or shame) and the safety threshold (A, B, or C), with the derived dose choice (standard or graded). A codebook defines operational criteria and boundary conditions, including concrete physiological and linguistic markers for thresholds and brief examples for each node. Short, de-identified clips and transcripts serve as training and test sets; accompanying answer keys are established by expert consensus. Design and metrics. A multi-site, rater-blinded study uses 60 to 90 vignette clips stratified by structural level and node-by-threshold combinations. After a standardized 90-minute training, independent raters label node and threshold and propose an action class (for example, downregulate and protect positives near a C boundary). Agreement statistics include Cohen’s kappa or Gwet’s AC1 for node, weighted kappa or intraclass correlation for thresholds, and decision-distance for action selection. *A priori* benchmarks are set at substantial agreement for node, moderate-to-substantial for threshold, and at least 80% concordance for dose selection. These targets are justified by the maturity of process markers in the ISTDP outcome literature and meta-analyses (12, 13, 37, 38). Integrity (adherence and competence). An adherence index expresses the proportion of in-session decisions that both match the observed node and threshold and select the rule-consistent dose (for example, switch to graded work as tolerance narrows from B toward C, halt deepening at C, and protect positive affect before any renewed confrontation). A brief competency rubric rates safety-critical behaviors and alliance preservation. This mirrors training trials and protocols in psychotherapy education that emphasize deliberate practice and structured feedback to improve interpersonal micro-skills (35, 36).

### 7.4 Patient-level outcomes and candidate mechanisms

*Primary outcomes*. Safety is defined as the frequency of C-level events per hour of active work and the mean time to recovery into the workable window. Alliance is assessed using a validated alliance measure and session-level rupture-repair counts. The directional hypotheses are that CSA-informed dosing reduces the rate of C exceedances and shortens recovery and that explicit protection of positive affect after progress stabilizes alliance trajectories (12, 13, 37, 38). Secondary outcomes (function). Each episode is linked to two to four Mini-ICF-APP domains (for example, endurance and persistence, planning and structuring, assertiveness, and dyadic relatedness) with re-check intervals tailored to the structural level. Primary functional indices are attainment of planned targets by the next review point and the slope of domain scores over time. The mechanistic hypothesis is that threshold-aware dosing yields faster functional gains because it minimizes destabilization while consolidating micro-progressions. Design. A pragmatic, stepped-care trial compares CSA-guided care with usual care, stratified by ICD-11 severity bands and personality impairment. Co-primary endpoints are the reduction in C-level events and improved alliance. A key secondary endpoint is the proportion of Mini-ICF-APP targets achieved at 6 to 8 weeks. Analyses use mixed-effects models with clinic as a random effect and baseline severity and structural level as covariates. Feasibility and expected effect directions are supported by ISTDP reviews and health-service outcomes, including cost and utilization signals in complex populations (12, 13, 37, 38). *Process checks*. Manipulation checks confirm that node-and-threshold labeling and dose selection differ between arms in line with CSA rules. The micro-episode sentence doubles as structured process data for audit and rater spot-checks.

### 7.5 Digital assist (HITL), not automation

*Rationale and scope*. The CSA’s value for computational psychiatry lies in discrete, observable labels (node, threshold, and action) and natural-language episode lines, which are amenable to supervised learning. Assistive models can flag likely transitions from B to C, post-progress superego attacks, or pathway shifts in anxiety while keeping decisions with the clinician. Plausible signals include audio-derived prosody and respiratory cadence, language markers of global self-condemnation, and simple attentional proxies; these align with current fidelity and supervision initiatives that augment human judgment rather than replace it (32, 42). Data, labels, transparency. Seed annotations come from clinician-verified episode lines. Models must expose the features that triggered a flag and present prompts that mirror the clinical rule set (for example, protect positives and regulate before any further deepening). This matches the broader movement toward explainable and workflow-respecting decision support (32, 42). Training and quality assurance (QA) pathway. Skills-first adoption uses brief clips with immediate feedback to improve recognition of threshold transitions and superego rapid reversal after a positive moment, leveraging the evidence base for deliberate practice and structured feedback in therapist training (35, 36). *Governance*. Data minimization, explicit consent, local governance, and on-device feature extraction are required. No deployment proceeds without auditable logs linked to episode lines and a clinician-override default. Bridge. We close by synthesizing contributions, limitations, and future work. Data registry (assistive, human-in-the-loop). Each episode is recorded as a compact, reproducible unit comprising the decision node, the active anxiety pathway and threshold within the window of tolerance, the presence of superego attack, the chosen action (standard or graded dosing with safety rules), and the resulting Mini-ICF-APP target with reassessment interval. This field set provides clean labels for supervised, clinician-governed analytics and mirrors the minimal schema specified in **Supplementary Material S1**.

### 7.6 Active inference bridges and falsifiable predictions

Active inference formalizes psychotherapy as iterated perception–action cycles in which agents sample the interpersonal environment to reduce uncertainty and update beliefs. The CSA instantiates this at the bedside: thresholding governs viable sampling, graded exposure near the B boundary controls precision of updates, and protection of positive states preserves adaptive priors after successful learning. This mapping supports specific predictions: graded dosing near the B boundary reduces epistemic uncertainty more efficiently than unregulated deepening, explicit protection of positive affect increases the persistence of adaptive priors and the density of progression steps, and externalizing punitive self-talk reduces maladaptive precision on destructive priors and improves assertiveness. These are testable within the designs outlined above and sit comfortably within contemporary formulations of predictive processing in clinical contexts (26, 39). Summary. The CSA’s compact rule set, observable thresholds, and functional bridge enable a coherent validation program: reliable node-and-threshold labeling, safer sessions with fewer crossings into C, a more stable alliance, measurable gains in Mini-ICF-APP targets, and transparent HITL augmentation. The program is deliberately pragmatic: it supports training and supervision now, aligns with dimensional nosology and functioning frameworks, and offers clean labels for careful computational work, while avoiding premature standardization. Evidence from ISTDP meta-analyses and service outcomes justifies clinical plausibility, training science supports the skills pathway, and active inference provides a principled account of why graded dosing and protection of positives should work when implemented faithfully (12, 13, 26, 32, 35–39, 42).

## 8 Discussion

### 8.1 Synthesis and contribution

This article specifies the CSA as a compact, teachable scaffold for moment-to-moment clinical decisions that is compatible with contemporary nosology, functioning language, and computational workflows. The core contribution is a small set of observable nodes—defense, anxiety/affect tolerance, progression, and superego/shame—coupled to three safety thresholds that gate intervention dose. Expressed entirely in prose, the rules ask the clinician to identify what is in front of the system, check the current threshold, match the dose to tolerance, protect positive affect when it appears, and then document the micro-episode in functional terms that a multidisciplinary team can use. The virtue of the approach is decision economy: a few simple rules organize a complex interaction space without flattening clinical nuance, while remaining measurable for teaching and audit. Evidence from ISTDP supports the plausibility of these rule families across diagnoses and service contexts, providing a clinical precedent for a threshold-sensitive dosing logic (13). The framework also clarifies distinctions that are often blurred in practice and training: structural defenses versus in-session resistance; shame, guilt, and self-accusation within superego pathology; and the difference between low observed anxiety and genuine affect tolerance. Teaching materials in contemporary ISTDP provide convergent psychodiagnostic scaffolds—resistance bands, pathway-based anxiety markers, and explicit stop rules for cognitive-perceptual disruption—that the CSA renders as a jargon-light, school-neutral documentation routine (anchored in observable markers and functional targets, without claiming theory-neutrality). This is compatible with other process-based, interpersonal training formulations (e.g., schema therapy) (52). It can also be translated into modality-specific language outside psychodynamic schools (e.g., avoidance and safety behaviors as defenses, physiological arousal/dissociation risk as anxiety/affect tolerance, approach behavior as progression, and self-criticism as superego/shame), while retaining the same safety constraints on dose and stop rules. Finally, the CSA complements established psychotherapy process and fidelity coding systems (e.g., otivational Interviewing Skill Code (MISC), Psychotherapy Process Q‑Set (PQS), Core Conflictual Relationship Theme (CCRT), and cognitive behavioral therapy (CBT) competence/process ratings) by targeting a different layer: threshold-controlled dosing decisions rather than intervention taxonomies or global technique adherence (53–56). This mapping preserves clinical craft while making the decision points legible for supervision and reliability studies (25, 29). A second contribution is a working bridge from diagnosis to functioning. Because each micro-episode closes with a functional impact statement in Mini-ICF-APP terms, the same observations that guide dosing also justify care intensity, inform stepped-care transitions, and define audit-ready goals (for example, endurance/persistence, planning/structuring, and dyadic relatedness). This translation helps explain why identical ICD-11 codes can warrant different resources: structural capacity and failure points under load diverge, and the CSA makes those divergences explicit in everyday language that teams already use (1, 2, 22). Finally, the algorithm’s sequence is naturally interpretable within active-inference accounts of clinical interaction. Small, safe prediction errors—delivered under threshold control—support learning about the patient’s generative model, protecting positive states stabilizes adaptive updates, and stopping at cognitive-perceptual disruption prevents catastrophic belief revisions. This alignment is not merely rhetorical: it motivates concrete, testable predictions about dosing, alliance stability, and the persistence of adaptive priors, and it explains why the CSA can be labeled and audited without turning the encounter into a checklist (39).

### 8.2 Limitations

The CSA is a theoretical and algorithmic proposal with a worked clinical illustration; it is not yet a validated clinical instrument. Its apparent simplicity can invite premature standardization. The present manuscript offers decision logic and a single composite case; it does not establish inter-rater reliability, sensitivity to change, or outcome effects. Those require a staged program of empirical work (13). Live, in-session use must remain contingent on thresholding and alliance. In fragile structures, attempts to deepen affect without dose control can trigger cognitive-perceptual disruption or shame collapse. The model therefore recommends a training-first deployment curriculum-integrated skills practice, video-based supervision, and service-level quality review—with cautious, context-sensitive use at the bedside. Existing psychodiagnostic frameworks that index dose to resistance level and anxiety pathways support this conservative stance (25, 29, 34). Digital augmentation is optional and must remain human-in-the-loop. Because the CSA labels are compact and observable, audio-lexical signals could, in principle, flag an approach to the upper threshold or a post-progress superego attack. However, any assistive analytics should surface interpretable features, respect workflow, and never dictate dosing. Protocol work in therapist-fidelity feedback illustrates how supportive automation can be designed without black-box prescriptions; the CSA’s labeled episodes are well suited to that paradigm if sites pursue it under governance and consent (32). Generalizability depends on teachability. The approach presumes that clinicians can learn to detect nodes and thresholds with reliability. Early training science suggests that deliberate practice with structured feedback improves interpersonal micro-skills, but effects vary by design and dosage. A CSA-specific curriculum and rater pack are therefore required before broader claims are warranted (35).

### 8.3 Future work

Three lines of inquiry are feasible and complementary. First, reliability: short, de-identified clips stratified by structural level can be used to train and test inter-rater agreement on node and threshold calls and to examine adherence to the dosing logic (for example, graded work near the upper threshold; immediate protection of positive affect after progress-linked superego attack). Second, outcomes: pragmatic trials in stepped-care programs can test whether CSA-guided work reduces the frequency and duration of cognitive-perceptual disruptions, stabilizes alliance, and accelerates attainment of Mini-ICF-APP goals relative to usual care or non-CSA supervision. Third, digital assist: where institutions opt in, interpretable, clinician-facing prompts can flag paralinguistic and lexical markers of threshold drift or punitive self-talk; all suggestions should remain advisory and auditable. Together, these studies would connect teachability, safety, alliance, and functioning in a single validation stream (32, 35, 39). Conceptually, active-inference modeling suggests specific predictions that can be falsified: graded dosing near the upper threshold should reduce epistemic uncertainty with fewer destabilizing events; explicit protection of positive states should increase the persistence of adaptive priors; externalizing the punitive voice should reduce the precision of self-attacking beliefs and support assertive action. These predictions align with the CSA’s prose logic and can be operationalized with the same episode labels used for training and QA (39). Conclusion. Psychotherapy can be conducted as a teachable state-space procedure expressed entirely in prose. The Conflict-Square Algorithm organizes observation around four nodes and three thresholds, matches dose to tolerance, protects nascent positive states, and closes each micro-episode with a functional impact statement that teams can use. This decision grammar preserves clinical nuance while making choices auditable and trainable. It is compatible with dimensional nosology and Mini-ICF-APP documentation, and it aligns with active-inference views of therapy as iterated perception–action under uncertainty. The path forward is pragmatic: establish reliability, test safety and alliance effects, evaluate functional gains, and, where appropriate, add human-in-the-loop assistive prompts. In doing so, the proposal connects mechanisms to metrics to services and offers a credible, testable contribution to computational psychiatry without overclaiming (13, 32, 35, 39). In addition, optional physiological monitoring (e.g., heart rate or heart rate variability from consumer wearables, used as biofeedback) could complement observable autonomic markers and support empirical threshold calibration, provided that consent, privacy, and clinical oversight are ensured.

## Data Availability

The original contributions presented in the study are included in the article/**Supplementary Material**. Further inquiries can be directed to the corresponding author.

## Appendix A: Interpretive map (active-inference lens)

Therapy can be read as iterated perception–action under uncertainty. Sampling: explicit A–C thresholds define viable sampling and prevent catastrophic updates. Learning: seconds-long graded exposures near a B boundary maximize information gain while preserving alliance; dose is widened only after stability is demonstrated. Precision and positives: newly adaptive states (for example, pride or closeness) require brief protection to stabilize precision before any renewed deepening; punitive dynamics are externalized and limited. Labels for HITL: because episodes are rendered in words (node, threshold, action, and Mini-ICF impact), consented pipelines can flag likely boundary crossings or post-progress punitive attacks without dictating care. This interpretive lens clarifies why the bedside rules improve safety and teachability while keeping jargon outside the main flow. (Cross-refs: Sections 1.2, 3.2–3.4, 4.7).

## References

[1] American Psychiatric Association . Diagnostic and Statistical Manual of Mental Disorders. 5th ed. Washington (DC: American Psychiatric Association (2013).

[2] American Psychiatric Association . DSM-5 Section III: Assessment Measures and the Alternative Model for Personality Disorders. Arlington, VA: American Psychiatric Association (2013).

[3] Seemüller F . ICD-11 and mental disorders: Important changes, controversies, and future directions. BMC Psychiatry. (2023) 23:698. doi: 10.1186/s12888-023-05186-w, PMID: 37749513 PMC10521444

[4] Skodol AE Morey LC Bender DS Oldham JM . The alternative DSM-5 model for personality disorders. Am J Psychiatry. (2015) 172:690–3. doi: 10.1176/appi.ajp.2015.14101220, PMID: 26130200

[5] Goodkind M Eickhoff SB Oathes DJ Jiang Y Chang A Jones‑Hagata LB . Identification of a common neurobiological substrate for mental illness. JAMA Psychiatry. (2015) 72:305–15. doi: 10.1001/jamapsychiatry.2014.2206, PMID: 25651064 PMC4791058

[6] Linden M Keller L Noack N Muschalla B . Self‑rating of capacity limitations in mental disorders: The “Mini‑ICF‑APP‑S”. Behavioral Medicine and Rehabilitation Practice. (2018) 101:14–22.

[7] Jaeger S Uhlmann C Bichescu-Burian D Flammer E Steinert T Schmid P . One-year follow-up of functional impairment in inpatients with mood and anxiety disorders- Potentials of the Mini-ICF-APP. BMC Psychiatry. (2022) 22:334. doi: 10.1186/s12888-022-03977-1, PMID: 35570275 PMC9107757

[8] Molodynski A Linden M Juckel G Yeeles K Anderson C Vazquez-Montes M . The reliability, validity, and applicability of an English language version of the Mini-ICF-APP. Soc Psychiatry Psychiatr Epidemiol. (2013) 48:1347–54. doi: 10.1007/s00127-012-0618-6, PMID: 23080483

[9] Pinna F Fiorillo A Tusconi M Guiso B Carpiniello B . Assessment of functioning in patients with schizophrenia and schizoaffective disorder with the Mini-ICF-APP: A validation study in Italy. Int J Ment Health Syst. (2015) 9:37. doi: 10.1186/s13033-015-0030-x, PMID: 26526168 PMC4628277

[10] Rosburg T Kunz R Trezzini B Schwegler U Jeger J . The assessment of capacity limitations in psychiatric work disability evaluations by the social functioning scale Mini-ICF- APP. BMC Psychiatry. (2021) 21:480. doi: 10.1186/s12888-021-03467-w, PMID: 34592979 PMC8485557

[11] Egger ST Weniger G Bobes J Seifritz E Vetter S . Exploring the factor structure of the Mini‑ICF‑APP in a clinical inpatient sample according to the main psychiatric diagnosis. Rev Psiquiatr Salud Ment. (2021) 14:186–95. doi: 10.1016/j.rpsmen.2021.11.002, PMID: 34861928

[12] Abbass A Kisely S Town J . Short-term psychodynamic psychotherapy for somatic disorders: Systematic review and meta-analysis of clinical trials. Psychother Psychosom. (2009) 78:265–74. doi: 10.1159/000228247, PMID: 19602915

[13] Abbass A Town J Driessen E . Intensive short-term dynamic psychotherapy: A systematic review and meta-analysis of outcome research. Harv Rev Psychiatry. (2012) 20:97–108. doi: 10.3109/10673229.2012.677347, PMID: 22512743

[14] Abbass A Town JM . Intensive short-term dynamic psychotherapy for complex, chronic, and treatment-resistant conditions. Am J Psychother. (2025) 78:160–6. doi: 10.1176/appi.psychotherapy.20240024, PMID: 39876702

[15] Fosha D . Undoing Aloneness & the Transformation of Suffering into Flourishing: AEDP 2.0. New York: The AEDP Institute Press (2021).

[16] Frederickson J . Co-Creating Change: Effective Dynamic Therapy Techniques. Washington, DC: Seven Leaves Press (2013).

[17] Frederickson J . Co-Creating Safety: Healing the Fragile Patient. Washington, DC: Seven Leaves Press (2020).

[18] Frederickson J . Healing Through Relating: A Skill-Building Book for Therapists. MD, USA: Seven Leaves Press (2023). Available online at: https://istdpinstitute.com/healing-through-relating/

[19] Friston KJ . The free-energy principle: A unified brain theory? Nat Rev Neurosci. (2010) 11:127–38. doi: 10.1038/nrn2787, PMID: 20068583

[20] Parr T Pezzulo G Friston KJ . Active Inference: The Free Energy Principle in Mind, Brain, and Behavior. Cambridge, MA: MIT Press (2022).

[21] Niederlohmann E . From Symptoms to Function: The PAD-S Decision Matrix—A Transdiagnostic Psychotherapy Algorithm for Severe Mental Illness. Geneva, Switzerland: Zenodo (2026). doi: 10.5281/zenodo.18504363, PMID: PMC13370903 42459285

[22] Bach B Brown TA Mulder RT Newton-Howes G Simonsen E Sellbom M . Development and initial evaluation of the ICD-11 Personality Disorder Severity scale (PDS-ICD-11). Pers Ment Health. (2021) 15:223–36. doi: 10.1002/pmh.1510, PMID: 34002530

[23] Pan B Chakhssi F Bastiaens T De Fruyt F . Practical implications of ICD-11 personality disorder severity. BMC Psychiatry. (2024) 24:56. doi: 10.1186/s12888-024-05640-3, PMID: 38454364 PMC10921591

[24] Kandel ER Koester JD Mack SH Siegelbaum SA . Principles of Neural Science. 6th ed. New York: McGraw-Hill (2021).

[25] Michal M Osborn K . Die Affektphobietherapie: Psychodynamisches transdiagnostisches manualisiertes Behandlungsmodell. Psychotherapeut. (2021) 66:314–23. doi: 10.1007/s00278-021-00490-w, PMID:

[26] Adams RA Friston KJ . Active inference and auditory hallucinations. Comput Psychiatry. (2018) 2:183–204. doi: 10.1162/cpsy_a_00022, PMID: 30627670 PMC6317754

[27] Kirchner L Eckert AL Berg M . From broken models to treatment selection: Active inference as a tool to guide clinical research and practice. Clin Psychol Eur. (2022) 4:e9697. doi: 10.32872/cpe.9697, PMID: 36397948 PMC9667420

[28] Knolle F Sterner E Moutoussis M Adams RA Griffin JD Haarsma J . Action selection in early stages of psychosis: An active inference approach. J Psychiatry Neurosci. (2023) 48:E78–89. doi: 10.1503/jpn.220141, PMID: 36810306 PMC9949875

[29] Lin B Bouneffouf D Landa Y Jespersen R Corcoran C Cecchi G . COMPASS: Computational mapping of patient-therapist alliance from session language. NPJ Ment Health Res. (2025) 15:166. doi: 10.1038/s41398-025-03379-3, PMID: 40374613 PMC12081631

[30] Ryu J Heisig S McLaughlin C Katz M Mayberg HS Gu X . A natural language processing approach reveals first-person pronoun usage and non-fluency as markers of therapeutic alliance in psychotherapy. iScience. (2023) 26:106860. doi: 10.1016/j.isci.2023.106860, PMID: 37255661 PMC10225921

[31] Solms M . The Hidden Spring: A Journey to the Source of Consciousness. London: Profile Books (2021).

[32] Creed TA Salama L Slevin R Tanana M Imel Z Narayanan S . Enhancing the quality of cognitive behavioral therapy in community mental health through artificial intelligence generated fidelity feedback (Project AFFECT): A study protocol. BMC Health Serv Res. (2022) 22:1177. doi: 10.1186/s12913-022-08519-9, PMID: 36127689 PMC9487132

[33] Deuschle M Scheydt S Hirjak D Borgwedel D Erk K Hennig O . Track treatment in psychiatry: the CIMH track model to overcome sector boundaries. Nervenarzt. (2020) 91:50–6. doi: 10.1007/s00115-019-0704-8, PMID: 30941457

[34] Korezelidou A Welte A Oster A Mahler L . Overcoming the lack of alternatives: Changes in the use of coercive measures after implementation of the recovery-oriented ‘Weddinger Modell’ in acute psychiatric care. J Psychiatr Res. (2025) 181:405–10. doi: 10.1016/j.jpsychires.2024.11.032, PMID: 39657565

[35] Berning A Sell S Andersen W Strauvß B Taubner S . Effects of deliberate practice and structured feedback in psychotherapy training (DeeP): Study protocol of a randomized controlled trial. BMC Psychol. (2024) 12:719. doi: 10.1186/s40359-024-02015-x, PMID: 39633501 PMC11616299

[36] Chow DL Miller SD Seidel JA Kane RT Thornton JA Andrews WP . The role of deliberate practice in the development of highly effective psychotherapists. Psychotherapy. (2015) 52:337–45. doi: 10.1037/pst0000015, PMID: 26301425

[37] Abbass A Bernier D Kisely S Town J Johansson R . Sustained reduction in healthcare costs after adjunctive graded ISTDP in psychotic disorders. Psychiatry Res. (2015) 228:538–43. doi: 10.1016/j.psychres.2015.05.056, PMID: 26106054

[38] Abbass A Kisely S Town J . Cost-effectiveness of ISTDP trial therapy. Psychother Psychosom. (2018) 87:255–6. doi: 10.1159/000487600, PMID: 29635230

[39] Cheadle JE Davidson-Turner KJ Goosby BJ . Active Inference and Social Actors: Towards a neuro-bio-social theory of brains and bodies in their worlds. Kölner Z Soziol. (2024) 76:317–50. doi: 10.1007/s11577-024-00936-4, PMID: 39429464 PMC11485288

[40] Abbass A Haghiri B . Intensive short-term dynamic psychotherapy for functional somatic disorders: a scoping review. Clin Neuropsychiatry. (2025) 22:111–20. doi: 10.36131/cnfioritieditore20250201, PMID: 40401139 PMC12090373

[41] Abbass A . Reaching through resistance: advanced psychotherapy techniques. MD, USA: Seven Leaves Press (2015).

[42] Cioffi V Ragozzino O Mosca LL Moretto E Tortora E Acocella A . Can AI technologies support clinical supervision? Assessing the potential of ChatGPT. Informatics. (2025) 12:29. doi: 10.3390/informatics12010029, PMID: 41725453

[43] Bayer S Bröcker AL Stuke F Just S Bertram G Grimm I . Level of structural integration in schizophrenia and schizoaffective disorders-Applicability and clinical associations. Front Psychiatry. (2024) 15:1388478. doi: 10.3389/fpsyt.2024.1388478, PMID: 38911709 PMC11192590

[44] Bröcker AL Bayer S Shai D Laubenstein S Stuke F Just S . Levels of structural integration mediate the impact of metacognition on functioning in non-affective psychosis: adding a psychodynamic perspective to the metacognitive approach. Front Psychol. (2020) 11:269. doi: 10.3389/fpsyg.2020.00269, PMID: 32153475 PMC7047329

[45] Gutiérrez F Aluja A Rodríguez C Gárriz M Peri JM Gallart M . Severity in the ICD-11 personality disorder model: Evidence from the PDS-ICD-11. Front Psychiatry. (2022) 13:1015489. doi: 10.3389/fpsyt.2022.1015489, PMID: 36699492 PMC9868964

[46] Jantzi C Moulin V . The ICD-11 personality disorder diagnosis in forensic psychiatry: Practical implications. Front Psychiatry. (2025) 16:1630512. doi: 10.3389/fpsyt.2025.1630512, PMID: 40904569 PMC12401950

[47] Lorentzen HS Wilberg T Hummelen B Ulberg R Kvarstein EH . Reliability and validity of the Personality Disorder Severity ICD-11 (PDS-ICD-11) scale. Pers Ment Health. (2024) 18:e1629. doi: 10.1002/pmh.1630, PMID: 39049719

[48] Hualparuca-Olivera L . Convergence between ICD-11 and AMPD dimensional personality disorder models: A systematic review. Front Psychiatry. (2023) 14:1325583. doi: 10.3389/fpsyt.2023.1325583, PMID: 38098639 PMC10719945

[49] Frederickson J . Treatment Resistance: The Addict who had “no problem” [psychotherapy training video/DVD]. Washington, DC, USA: ISTDP Institute (2025). Available online at: https://istdpinstitute.com/dvds/ (Accessed March 17, 2025).

[50] Frederickson J . Restructuring Projection in a Borderline Patient: Instructional VideoxBundle [psychotherapy training video bundle]. Washington, DC, USA: ISTDP Institute (2025). Available online at: https://istdpinstitute.com/dvds/ (Accessed March 17, 2025).

[51] Frederickson J . Treatment of the Fragile Patient [psychotherapy training video/DVD]. Washington, DC, USA: ISTDP Institute (2025). Available online at: https://istdpinstitute.com/dvds/ (Accessed March 17, 2025).

[52] Roediger E Valente M . Schematherapie: Kontextuell - prozessbasiert - interpersonal. Stuttgart: Schattauer (2025).

[53] Miller WR Moyers TB Ernst D Amrhein P . Manual for the Motivational Interviewing Skill Code (MISC). Version 2.0. Albuquerque, NM: Center on Alcoholism, Substance Abuse and Addictions, The University of New Mexico (2003). Available online at: https://motivationalinterviewing.org/sites/default/files/MISC2.pdf (Accessed January 11, 2026).

[54] Ablon JS Levy RA Smith-Hansen L . The contributions of the psychotherapy process Q-set to psychotherapy research. Res Psychother: Psychopathol Process Outcome. (2011) 14:14–48. doi: 10.4081/ripppo.2011.46, PMID: 41149044

[55] Luborsky L Crits-Christoph P . Understanding Transference: The Core Conflictual Relationship Theme Method. New York: Basic Books (1990).

[56] Vallis TM Shaw BF Dobson KS . The Cognitive Therapy Scale: psychometric properties. J Consult Clin Psychol. (1986) 54:381–5. doi: 10.1037//0022-006X.54.3.381, PMID: 3722567

