# Supplementary material for: A transdiagnostic conflict-square algorithm: a four-node computational framework for psychotherapy and functional diagnosis
Source: Front Psychiatry. 2026 Mar 16;17:1687372. doi: 10.3389/fpsyt.2026.1687372 (PMC13033735; doi:10.3389/fpsyt.2026.1687372)
Supplement: Supplementary file 1 [file DataSheet1.docx]

**Supplementary Data Sheet 1 (S1)**

**Conflict Square Algorithm (CSA): Step-by-Step Specification, Decision Rules, and Pseudocode**

Related to: A Transdiagnostic Conflict Square Algorithm: A Four Node Computational Framework for Psychotherapy and Functional Diagnosis (Manuscript ID: 1687372)

Eik Niederlohmann

**S1.1 Scope and relation to main article**

This supplement formalizes the Conflict Square Algorithm (CSA) as a teachable, auditable procedure. It expands the prose description in the main text with compact decision rules, safety thresholds, micro-exposure drills, and a documentation grammar. The specification is school-agnostic and compatible with ICD-11 severity statements and Mini-ICF-APP outputs.

Reader note: This supplement provides an auditable training and documentation scaffold; it is not a validated standalone instrument and it is not intended for automated diagnosis or treatment decisions.

**S1.2 Observable nodes (front-of-system)**

Defense (DEF): identifiable maneuvers that divert from authentic affect or wish; tactical versus character patterns.

Anxiety/Affect Tolerance (ANX): physiological and cognitive load signals across three pathways (striated, smooth muscle, cognitive-perceptual) with a trend over time.

Progression (PRO): any forward relational movement (naming a wish, seconds of steady gaze, accepting help, value-consistent micro-action).

Superego/Shame (SUP): global self-attack and collapse following progress; includes destructive guilt and persecutory tones.

Node recognition anchors observable lexemes, posture, and prosody; thresholding uses pathway markers and coherence.

**S1.3 Thresholds and markers (A–C window)**

A: regulated arousal; symbolization intact; safe to deepen in short bouts.

B: narrowing yet workable range; micro-dose exposures; watch B→C drift.

C: cognitive-perceptual disruption or shame collapse; stop deepening; regulate; protect positives first.

Pathways: striated (A–B), smooth (B with B→C risk), cognitive-perceptual (C). Absence of overt anxiety does not imply high tolerance; stability under attachment-laden probes must be demonstrated.

**S1.4 Node × Threshold → Next action (compact rules)**

| Node \ Threshold | A (regulated) | B (working) | B→C (proximity) | C (disruption) |
| --- | --- | --- | --- | --- |
| Defense (DEF) | Name tactic; brief block; return to feeling/wish. | Block tactically; link defense → feeling → action. | Partial block; co-regulate; seconds-long exposure. | Stop deepening; regulate; postpone confrontation. |
| Anxiety/Affect Tolerance (ANX) | Proceed in short bouts; monitor markers. | Micro-dose; paced exhale; orienting; check markers. | Down-shift; co-regulate; abandon deepening; re-enter lower. | Stop; restore symbolization; safety first. |
| Progression (PRO) | Validate; link to tiny action; document. | Validate; brief amplification; tie to Mini-ICF target. | Buffer gains; consolidate in seconds; anticipate SUP. | Prioritize regulation/safeguard; do not pursue progression-deepening moves at C. |
| Superego/Shame (SUP) | Name punitive process; gentle limits; continue graded work. | Protect positives; externalize critic; compassion + limits; then proceed. | Protect positives first; no confrontation until A–B restored. | Protect; regulate; no confrontation; alliance repair. |

**S1.5 Stop rules and safety heuristics**

At threshold C, halt deepening and regulate before any renewed exposure.

When progress triggers a superego attack (joy → attack → collapse), protect positive affect first; externalize the punitive voice; pair compassion with limits; only then resume graded work.

Use seconds-long exposure windows near B→C; extend only after stability is demonstrated.

**S1.6 Seconds-long micro-exposure drills (graded format)**

Use 2–10-second windows with paced exhale, orienting, and explicit consent. Immediately check for smooth-muscle spikes or cognitive-perceptual signs. End each successful window by naming the step and predicting functional payoffs (e.g., more stable dyadic participation).

**S1.7 Documentation grammar (episode line)**

Template: Trigger → Observable response → Threshold (A–C) → Action → Mini-ICF-APP target and recheck interval.

Example 1: Direct praise → breath holding + head drop → B→C (shame) → protect positives; externalize critic; 2 s exposure → Dyadic relatedness: one protected appreciation exchange/week; recheck 4–6 weeks.

Example 2: Eye-contact trial → fogging/tunnel vision → C → stop deepening; regulate → Endurance: three grounding cycles/session; recheck 8–12 weeks.

**S1.8 Pseudocode (readable form)**

1. Identify front-of-system: DEF/ANX/PRO/SUP.
2. Estimate threshold: A vs. B vs. C using pathway markers.
3. Choose dose: standard if stable A–B; graded if B→C; stop at C.
4. If SUP follows PRO, protect positives first; externalize critic; add limits.
5. Execute micro-action; then record the episode line with a Mini-ICF target.
6. Repeat in short cycles; widen B safely; avoid repeated C events.

**S1.9 Reliability anchors and rater prompts**

Anchor Node calls to observable lexemes and posture; anchor Threshold to pathway and coherence. Use brief calibration sets and compute agreement (e.g., κ for Node; weighted κ for Threshold). Safety-critical behaviors include immediate down-regulation at C and protection of positives when SUP follows PRO.

**S1.10 Data registry (assistive, human-in-the-loop)**

Each episode is recorded as a compact unit: {node, threshold, anxiety_pathway, superego_attack (yes/no), action, mini_icf_domain, phrasing, recheck_weeks, safety_flag}. This field set supports supervised, clinician-governed analytics and mirrors the minimal schema in S1.
