# Supplementary material for: A transdiagnostic conflict-square algorithm: a four-node computational framework for psychotherapy and functional diagnosis
Source: Front Psychiatry. 2026 Mar 16;17:1687372. doi: 10.3389/fpsyt.2026.1687372 (PMC13033735; doi:10.3389/fpsyt.2026.1687372)
Supplement: Supplementary file 2 [file DataSheet2.docx]

**Supplementary Data Sheet 2 (S2)**

**Annotated Session Transcripts and Rater Pack**

Related to: A Transdiagnostic Conflict Square Algorithm: A Four Node Computational Framework for Psychotherapy and Functional Diagnosis (Manuscript ID: 1687372)

Eik Niederlohmann

**S2.1 Ethical and methodological note**

All illustrative sequences are composites; identifiers are removed or altered. Segments are 20–60 seconds long to support micro-decision training. Raters code Node, Threshold, and Action, then compose one episode line per segment.

**S2.2 Label grammar and coding fields**

Required fields: node (DEF/ANX/PRO/SUP); threshold (A/B/C); pathway (striated/smooth/cognitive-perceptual); action (standard/graded/stop); episode_line (Trigger → Response → Threshold → Action → Mini-ICF); safety_flag; notes.

**S2.3 Calibration protocol**

Training set: 12 clips stratified by structure and node/threshold mixture. Raters label independently, then reconcile using observable markers. Suggested starting targets: κ ≥ 0.70 for Node; ≥ 0.65 for Threshold. Compute decision distance for Action.

**S2.4 Annotation guide with exemplars**

Exemplar A — SUP whiplash after PRO.
Transcript: “I felt proud yesterday.” (voice brightens, 2–3 s gaze). Then: “Who am I kidding.” (head drop).
Code: node=SUP; threshold=B→C; pathway=smooth→cognitive-perceptual risk; action=protect positives; externalize critic; seconds-long exposure.
Episode line: Pride share → head drop + global self-attack → B→C risk → protect positives; externalize; 2 s exposure → Dyadic relatedness: one protected appreciation exchange/week; recheck 4–6 weeks.

Exemplar B — Low anxiety ≠ high tolerance.
Transcript: Therapist: “Hold my eyes for 10 seconds.” Patient: “Sure.” After 3 seconds, speech fragments and gaze tunnels.
Code: node=ANX; threshold=C; pathway=cognitive-perceptual; action=stop; regulate; postpone exposure.
Episode line: Eye-contact trial → fogging/tunnel vision → C → stop deepening; regulate → Endurance: three grounding cycles/session; recheck 8–12 weeks.

Exemplar C — Defense vs. resistance.
Transcript: Therapist: “What did you want to ask your manager?” Patient jokes and changes topic; posture steady.
Code: node=DEF; threshold=A–B; action=clarify and block; return to feeling; brief deepen.
Episode line: Ask rehearsal → joking detour → A–B → clarify/block; one sentence to feeling → Assertiveness: one graded request/week; recheck 6 weeks.

**S2.5 Rater worksheets and scoring**

Worksheet template (one per clip). Enter values using the allowed labels; keep episode lines concise and observable.

| Clip ID |  |
| --- | --- |
| Trigger |  |
| Observable response (markers) |  |
| Node (DEF/ANX/PRO/SUP) |  |
| Threshold (A/B/C) |  |
| Pathway (striated/smooth/CPD) |  |
| Dose/Action (standard/graded/stop) |  |
| Episode line (Trigger → Response → Threshold → Action → Mini-ICF) |  |
| Safety flag (yes/no; type) |  |
| Notes |  |

Use one sheet per clip; include binary safety items (C detected and stop applied; SUP after PRO and positives protected). Compute adherence to dose logic (graded near B→C; stop at C). Summarize individual and group κ with 95% CI.

**S2.6 Competence rubric (safety-critical behaviors)**

Detects C and halts deepening — exceeds standard: stops within 2 s; initiates co-regulation promptly. Minimum standard: stops within 10 s; regulates.

Protects positives after PRO→SUP — exceeds standard: names switch; externalizes; holds 20–30 s. Minimum standard: names switch; brief protection.

Dose near B→C — exceeds standard: windows ≤ 5 s; frequent check-ins. Minimum standard: windows ≤ 10 s; at least one check-in.

Defense vs. resistance — exceeds standard: differentiates and times confrontation precisely. Minimum standard: avoids confrontation when alliance is strained.

**S2.7 Alliance and safety event definitions**

Alliance micro-rupture: immediate drop in contact, compliance with contempt, or visible withdrawal. Safety event: any C-level sign or unprotected SUP after PRO. Repair: join before challenge; regulate; protect positives; only then resume graded work.
