# Supplementary material for: A transdiagnostic conflict-square algorithm: a four-node computational framework for psychotherapy and functional diagnosis
Source: Front Psychiatry. 2026 Mar 16;17:1687372. doi: 10.3389/fpsyt.2026.1687372 (PMC13033735; doi:10.3389/fpsyt.2026.1687372)
Supplement: Supplementary file 3 [file DataSheet3.docx]

**Supplementary Data Sheet 3 (S3)**

**Mini-ICF-APP Crosswalk and Phrase Bank**

Related to: A Transdiagnostic Conflict Square Algorithm: A Four Node Computational Framework for Psychotherapy and Functional Diagnosis (Manuscript ID: 1687372)

Eik Niederlohmann

**S3.1 Purpose and scope**

This crosswalk translates CSA episode lines into team-ready Mini-ICF-APP phrasing with target behaviors and reassessment intervals. Entries are examples; teams should adapt to local formulations while preserving clarity and auditability.

**S3.2 How to use this crosswalk**

For each observed pattern (node/threshold/pathway), select one to two domains most impacted, adopt a copy-ready sentence, and specify a review window consistent with structural capacity. Use conservative intervals in fragile presentations (8–12 weeks) and shorter intervals in robust presentations (4–6 weeks).

**S3.3 Pattern → domain mapping (examples)**

| CSA pattern | Likely domains | Copy-ready phrasing + Recheck |
| --- | --- | --- |
| Attachment cue triggers B→C (smooth-muscle spike) | Endurance; planning/structuring; dyadic relatedness | Under attachment-laden demand, endurance and planning may decrease as B→C approaches. We will titrate proximity in 5–10 s windows with co-regulation and scaffold a three-step plan-and-do task. Recheck: 6–8 weeks. |
| PRO followed by SUP (joy→attack→collapse) | Assertiveness; dyadic relatedness; enjoyment | Positive states require protection to consolidate. We will rehearse one appreciation exchange weekly and hold the positive state for 20–30 s before moving on. Recheck: 4–6 weeks. |
| Calm surface with CPD at minimal load (C) | Endurance; group interaction; decision making | At minimal relational demand, cognitive–perceptual signs appear. We will limit exposure to seconds and implement a staff-wide shame-repair script. Recheck: 8–12 weeks. |
| Tactical detours at A–B with stable symbolization | Planning/structuring; assertiveness | Detours reduce planning quality. We will clarify and return to one-sentence feeling, then rehearse one graded request per week. Recheck: 6 weeks. |
| Humiliation trigger with contempt and rapid SUP | Rule adherence; dyadic relatedness | Under humiliation cues, rule adherence may destabilize. We will pre-commit to a two-step script and rehearse it in vivo before group participation. Recheck: 4–6 weeks. |

**S3.4 Phrase bank by domain**

Endurance/persistence: Practice three grounding cycles daily and during sessions when B→C signs appear; aim for steadier effort; reassess in 6–8 weeks.

Planning/structuring: Use a three-step checklist for attachment-laden tasks; supervisor reviews weekly; aim for fewer detours and clearer sequencing; reassess in 6–8 weeks.

Decision making: After regulation, complete two-step decisions with brief coaching; monitor for improvement over 4–6 weeks.

Assertiveness: One value-consistent graded request per week at work and home; therapist protects positives immediately after success.

Dyadic relatedness: Tolerate 10–15 seconds of eye contact with co-regulation; one protected appreciation exchange per week.

Group interaction/rule adherence: Rehearse a pre-commitment script before groups; maintain participation for 10–15 minutes post-trigger; supervisor checks adherence.

**S3.5 Severity-tiered intervals**

Robust A–B: 4–6 weeks. Mixed structure with B→C risk: 6–8 weeks. Fragile with C at minimal load: 8–12 weeks.

**S3.6 Worked examples**

Example 1: Direct focus on manager conflict → isolation + striated tension → B→C with smooth-muscle spike; later joy→attack pattern → protect positives; externalize; seconds-long exposure only → Likely reduced endurance and oscillating dyadic relatedness under dyadic demand; recheck 6–8 weeks.

Example 2: Specific ask formulated → stable breath/voice → Threshold B → validate; rehearse; protect positives → Dyadic relatedness at work: one graded request/week; recheck 4–6 weeks.
