# Supplementary figures and images for: A transdiagnostic conflict-square algorithm: a four-node computational framework for psychotherapy and functional diagnosis
Source: Front Psychiatry. 2026 Mar 16;17:1687372. doi: 10.3389/fpsyt.2026.1687372 (PMC13033735; doi:10.3389/fpsyt.2026.1687372)

PAD-S micro-decision loop (Perceive – Assess – Dose – Safeguard)

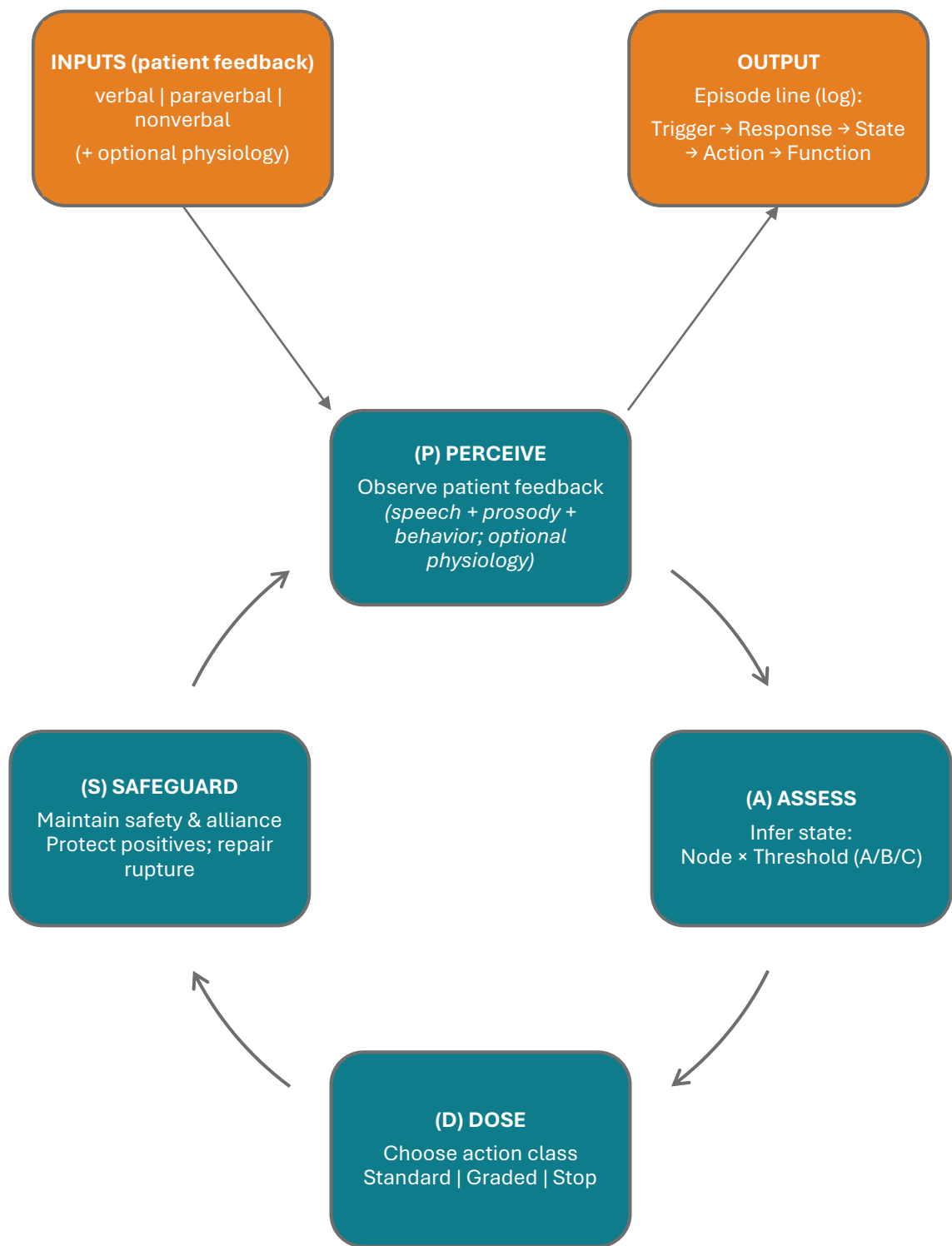

Supplement: Supplementary file 4 [file DataSheet4.pdf]
